# Fasting increases microbiome-based colonization resistance and reduces host inflammatory responses during an enteric bacterial infection

**DOI:** 10.1371/journal.ppat.1009719

**Published:** 2021-08-05

**Authors:** Franziska A. Graef, Larissa S. Celiberto, Joannie M. Allaire, Mimi T. Y. Kuan, Else S. Bosman, Shauna M. Crowley, Hyungjun Yang, Justin H. Chan, Martin Stahl, Hongbing Yu, Candice Quin, Deanna L. Gibson, Elena F. Verdu, Kevan Jacobson, Bruce A. Vallance

**Affiliations:** 1 Department of Pediatrics, BC Children’s Hospital, University of British Columbia, Vancouver, British Columbia, Canada; 2 Department of Biology, University of British Columbia, Kelowna, British Columbia, Canada; 3 Farncombe Institute, McMaster University, Hamilton, Ontario, Canada; University of California Davis School of Medicine, UNITED STATES

## Abstract

Reducing food intake is a common host response to infection, yet it remains unclear whether fasting is detrimental or beneficial to an infected host. Despite the gastrointestinal tract being the primary site of nutrient uptake and a common route for infection, studies have yet to examine how fasting alters the host’s response to an enteric infection. To test this, mice were fasted before and during oral infection with the invasive bacterium *Salmonella enterica* serovar Typhimurium. Fasting dramatically interrupted infection and subsequent gastroenteritis by suppressing *Salmonella’s* SPI-1 virulence program, preventing invasion of the gut epithelium. Virulence suppression depended on the gut microbiota, as *Salmonella*’s invasion of the epithelium proceeded in fasting gnotobiotic mice. Despite *Salmonella*’s restored virulence within the intestines of gnotobiotic mice, fasting downregulated pro-inflammatory signaling, greatly reducing intestinal pathology. Our study highlights how food intake controls the complex relationship between host, pathogen and gut microbiota during an enteric infection.

## Author summary

Most animals, including humans, lose their appetites when sick. Whether this sickness behavior has evolved as a protective mechanism is unclear. In addition, fasting therapies have become popular in recent years and show promise for treating chronic inflammatory diseases, but it is uncertain whether fasting-induced immunosuppression could leave an already fasted host more vulnerable to infection than a fed host. To test this, we fasted mice and orally infected them with the invasive bacterium *Salmonella* Typhimurium. This pathogen causes gastroenteritis (food poisoning) in humans and in antibiotic-pretreated mice. Notably, the fasted mice were protected from infection. While *Salmonella* rapidly expanded in the intestines of fed mice, their expansion was reduced in fasted mice. Moreover, *Salmonella* in the fasted mice did not cause any intestinal tissue damage as the bacteria were unable to invade the intestinal wall. This protection was found to be partially due to the gut microbiome, since fasting was unable to prevent *Salmonella* infection in mice lacking a microbiome, although the mice suffered less gastroenteritis. We therefore conclude that fasting can protect hosts from intestinal bacterial infections, in part through the actions of the gut microbiome.

## Introduction

When a mammalian host becomes infected, concerted metabolic programs are activated at both the cellular and organ level to combat the invading pathogen, distributing energy resources away from growth and towards an effective immune response [1]. These internal metabolic adaptations are accompanied by external sickness behaviors such as fever, malaise, lethargy, social withdrawal and loss of appetite [2]. Intuitively, it would appear maladaptive for a host to reduce food intake (*i*.*e*. undergo fasting) at a time when a metabolically expensive immune response is required. Even so, decreased appetite following infection is a highly conserved behavior, suggesting that it may be host-protective [3].

Several studies support this hypothesis, including experiments from 1979 demonstrating that previously fasted mice showed greatly reduced mortality following infection with an intravenous (*i*.*v*.) dose of *Listeria monocytogenes* known to be lethal to fed mice [4]. Conversely, interrupting this natural fasting response by force-feeding infected mice dramatically increased their mortality rates [5]. In contrast, research has shown that the fasting response can prove highly detrimental in mice during systemic viral [6] and bacterial infections [7]. In fact, Rao *et al*. found that the bacterial pathogen *Salmonella enterica* serovar Typhimurium has potentially evolved adaptive counter measures, limiting systemic infection-induced anorexia at the cost of its own virulence, as a means to ensure host survival and thus its own transmission [7]. In line with these findings are observations from severely food deprived populations that malnutrition, especially protein deficiency, is the primary cause of immunodeficiency worldwide. Chronic undernutrition impairs both innate and adaptive immune responses, leading to the deaths of millions of children from otherwise non-lethal infections [8].

Following the concept that reducing nutrient intake suppresses immune function, significant attention has recently been paid to purposeful fasting as a novel treatment for chronic inflammatory diseases such as rheumatoid arthritis and multiple sclerosis [9,10]. These inflammatory conditions involve aberrant activation of the adaptive immune system, and correspondingly, fasting or consumption of a fasting-mimicking diet were found to reduce the number and activity of autoreactive T cells [10]. Similarly, prolonged fasting was found to reduce circulating monocytes in humans, as well as the emergency mobilization of monocytes in mice, in response to infection with *L*. *monocytogenes* [11].

It should be noted that many bacterial and viral pathogens target the mammalian gastrointestinal (GI) tract. As the primary site of nutrient acquisition, it is also the first organ to be affected by reduced food intake. Additionally, the GI tract contains a rich commensal microbial community that plays an important role in both nutrient uptake and host defense. At present, little is known regarding how fasting may affect the GI tract or its resident microbiota. While there is evidence that fasting elicits tissue-protective programs that leave the host less susceptible to pathogen-mediated immunopathology [6,12,13], it remains uncertain how this would function in the gut, and whether such immune suppression would impact intestinal host defense. Moreover, since enteric pathogens often rely on host and microbiota-derived nutrients for both energy and virulence cues [14], it remains to be tested how a pathogen would function upon entering the GI tract of a fasted host.

To address these questions, the bacterial pathogen *S*. Typhimurium was selected since its virulence properties are well characterized and its pathogenic strategy involves invasion of host cells, intimately exposing *S*. Typhimurium to its host’s metabolism. Rather than repeating earlier systemic infection studies [7], a model of infectious gastroenteritis, involving antibiotic-based disruption of microbiota-based colonization resistance was utilized. Upon reaching the cecum, orally gavaged *S*. Typhimurium transcribe several key virulence factors encoded within the *Salmonella* pathogenicity island (SPI)-1, enabling the pathogen to invade the intestinal epithelium. This invasion triggers severe cecal inflammation and mucosal pathology within 6 hours post-infection [15]. The rapid onset of infection and gastroenteritis in this model allowed us to assess the effects of fasting throughout the entire acute infection period.

Notably, we found that fasting blocked *S*. Typhimurium’s ability to invade the intestinal epithelium through suppression of SPI-1-based cellular invasion, thereby preventing gastroenteritis development. This occurred in a microbiota-dependent manner, since pathogen invasion of the epithelium still occurred in fasted, germfree mice. Interestingly, fasting abrogated the host inflammatory responses to infection in the germfree mice, through decreased activation of the pro-inflammatory transcription factor NF-κB, leading to greatly reduced pathology. Thus, acute fasting protects against pathogen-induced gastroenteritis through its effects on the gut microbiome, as well as by suppressing pathogen virulence and the host inflammatory response.

## Results

### Mice in a fasted state are protected from *S*. Typhimurium-induced gastroenteritis

In the *S*. Typhimurium model of infectious gastroenteritis, mice are pre-treated with the antibiotic streptomycin to overcome microbiota-based colonization resistance [16]. This antibiotic treatment is followed 24 hours (h) later by an oral gavage of 2.5 x 10^6^ colony forming units (CFU) of *S*. Typhimurium, which very rapidly infect the cecal epithelium, triggering inflammation and tissue pathology within 6h, and overt gastroenteritis by 24h [16]. Correspondingly, although mice possess a higher metabolic rate than humans, they can still fast for a period of 48h without compromising their health [17]. We therefore fasted C57BL/6 mice for this period, and verified metabolic markers of fasting. As expected, blood glucose levels were decreased (< 80 mg/dL) while serum ketone (β-hydroxybutyrate) levels were increased (>1.5 mmol). These parameters are all indicative of a robust fasted metabolic state in mice. Similarly, the fasted mice lost just under 20% of their body weight after the 48h fast. While body weight loss is often used as an indicator of disease activity, fasting-induced weight loss is not on its own reflective of diminished health [18]. Upon re-feeding, fasted-uninfected mice quickly regained weight [19,20]. Moreover, 48h fasted mice did not exhibit higher disease scores at 24h post infection (activity, appearance, posture (AAP), hydration) than fed-infected mice **(S1A–S1D Fig).**

**Fig 1:**
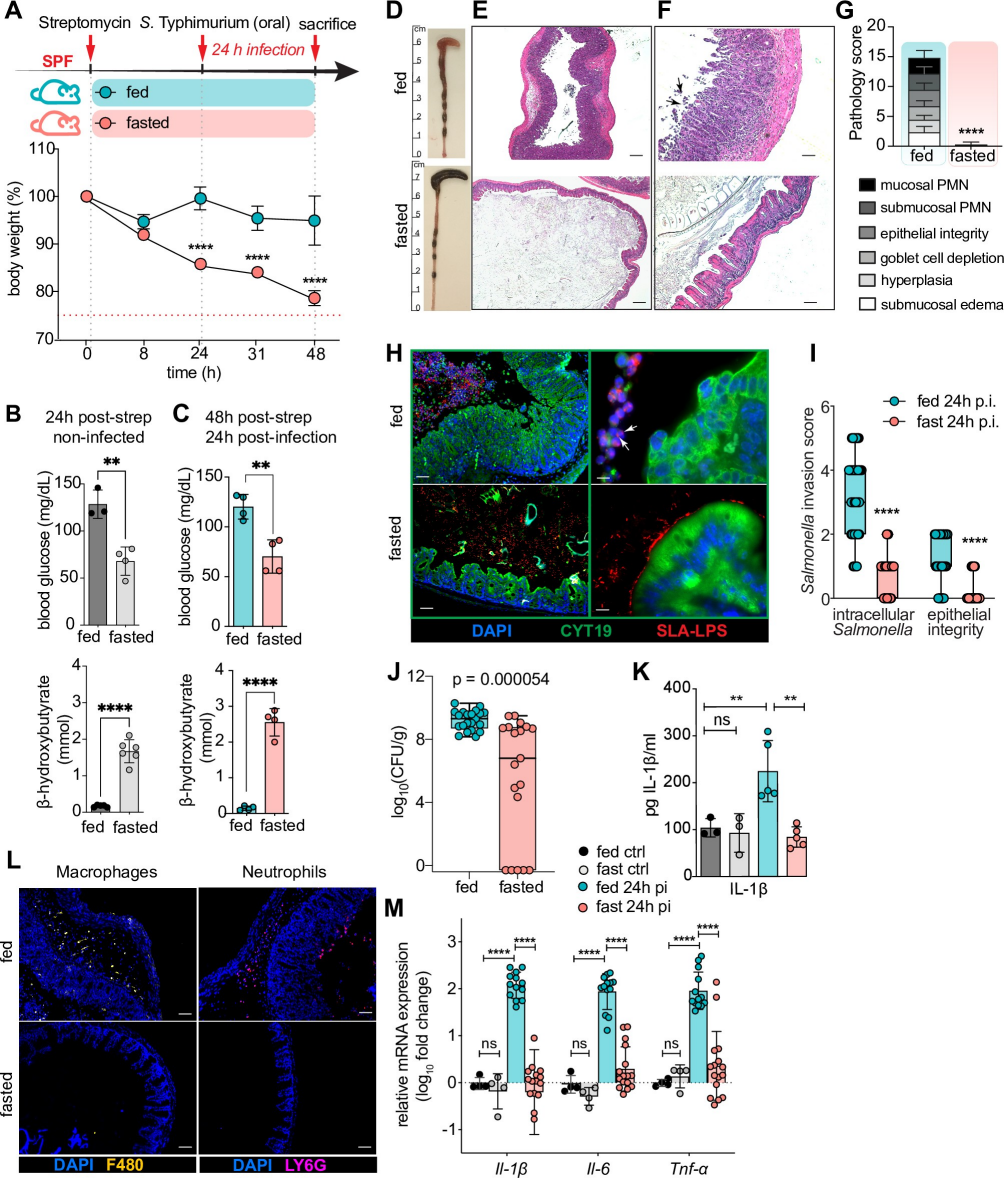
*S*. Typhimurium-induced gastroenteritis and accompanying inflammatory responses are abrogated by fasting and *S*. Typhimurium remains luminal in fasted mice. (A) Experimental timeline of infection and fasting regimen and concurrent body weight loss. Streptomycin-pretreated SPF (specific pathogen free) C57BL/6 mice were orally gavaged with ~ 2.5 × 10^6^ colony-forming units (CFU) of *S*. Typhimurium and sacrificed 24h p.i. (n = 5/group) (B) blood glucose and β-hydroxybutyrate levels in mouse serum at 24h post streptomycin treatment ± 24h of fasting. (C) blood glucose and β-hydroxybutyrate levels in mouse serum at 24h p.i. with *S*. Typhimurium ± 48h of fasting. (D) Representative macroscopic images of mouse cecal and colonic pathology 24h p.i. with *S*. Typhimurium ± 48h of fasting. (E-F) Representative hematoxylin-and-eosin (H&E)-stained cecal sections of mice at 24h p.i. with *S*. Typhimurium ± 48h of fasting. Arrows indicate sloughed epithelial cells. Scale bar 200 μm (E), 50 μm (F). (G) Histopathological analysis of cecal H&E tissue sections (as shown in (E), see Materials and Methods for scoring criteria). Agreement among raters ensured by Kendall’s coefficient of concordance Wt = 0.9033 (n ≥ 16). (H) Representative immunofluorescence staining of *S*. Typhimurium and IEC from cecal sections at 24h p.i. ± 48h of fasting. Sections were stained using DAPI to detect DNA (blue), anti-*Salmonella*-LPS (SLA, red) to visualize *S*. Typhimurium and anti-cytokeratin 19 (CYT19, green) to stain IEC. White arrows indicating sloughed IEC containing *Salmonella*. Scale bar 50 μm (left), 10 μm (right). (K) IL-1β protein levels measured by ELISA in whole cecum lysates of mice infected with *S*. Typhimurium at 24h p.i. ± 48h of fasting. Fed ctrl and fast ctrl refer to uninfected *ad libitum* fed or 48h fasted C57BL/6 SPF mice. (I) Invasion score quantifying *S*. Typhimurium presence in IEC by analyzing immunofluorescently stained cecal sections at 24h p.i. shown in (H) (see Materials and Methods for scoring criteria). Agreement among raters ensured by Kendall’s coefficient of concordance Wt = 0.861 (n ≥ 16). (J) *S*. Typhimurium CFU per g cecal tissue and stool (combined) 24h p.i. ± 48h of fasting. (L) Representative immunofluorescence staining of macrophages and neutrophils on cecal sections at 24h p.i. ± 48h of fasting. Sections were stained using DAPI to detect DNA (blue), mononuclear phagocyte marker anti-F4/80 (yellow) or neutrophil marker anti-Ly6G (pink). Scale bar 50 μm. (M) qPCR analysis of inflammatory genes in mouse cecum expressed as fold change over fed ctrl. All data shown repeated in at least 3 independent experiments. Mice with no detectable pathogen burdens were excluded from the analysis/images shown in D-I and K-M. For (G, I, J, M) data from multiple independent experiments were pooled. **** p < 0.0001, ** p < 0.01, ns = not significant. Significance levels calculated by unpaired Student’s t test (B, C, G), multiple t-test with Holm-Sidak correction (A), 1-way ANOVA (K), 2-way ANOVA with Tukey post-test (M), Mann-Whitney-Wilcoxon test (I, J) with Holm-Sidak correction. Error bars shown as ± SD except (I,J), which are shown as box plot with min-max whiskers. Related to S1 Fig.

To test the effects of fasting on *S*. Typhimurium-induced gastroenteritis, mice were fasted for the 24h post-streptomycin period, then infected with the streptomycin-resistant *S*. Typhimurium strain SL1344, with fasting continuing for the first 24h of infection (total fasting time 48h, total infection time 24h –see **Fig 1A**). This approach allowed us to assess how fasting alters pathogen-host interactions within the intestine. We confirmed that the fasted-infected mice had switched to fasting metabolism at the time of infection and had remained in this state at 24 h post infection **(Fig 1B and 1C)**. The fed-infected mice showed overt, infection-induced pathology, including a shrunken cecum, whereas the fasted-infected mice showed few macroscopic signs of intestinal damage **(Fig 1D)**. Consistent with this finding, there was a dramatic difference in cecal histology between fed and fasted-infected mice. Fed-infected mice suffered severe cecal pathology including widespread inflammatory cell infiltration into the mucosa and submucosa, crypt hyperplasia, edema as well as massive epithelial cell sloughing into the cecal lumen [16]. In contrast, fasted-infected mice showed minimal intestinal tissue damage. Their cecal epithelium remained completely intact and neither crypt hyperplasia nor edema were observed. Similarly, only a few immune cells were identified during histological assessment **(Fig 1E–1G).**

### Fasting abrogates the host pro-inflammatory response to *S*. Typhimurium infection

To further examine the host response to infection, immunofluorescence staining was performed to identify infiltrating inflammatory cells. Corresponding with their reduced histopathology, staining confirmed a markedly reduced inflammatory response in the fasted mice. While numerous infiltrating neutrophils (Ly6G+) and mononuclear phagocytes (F4/80+ macrophages, monocytes and dendritic cells) were present in the infected ceca of fed mice, there was no evidence of this response in fasted mice **(Fig 1L).** Similarly, analysis of inflammatory gene transcripts for the main pro-inflammatory cytokines *Il-1β*, *Il-6* and *Tnf-α* in cecal tissues showed the expected increase in the fed group, whereas this response was significantly attenuated in the fasted mice. This was also confirmed for cecal IL-1β protein levels **(Fig 1K and 1M).**

### *S*. Typhimurium remains luminal in the ceca of fasted mice

We next examined whether entering a fasted host attenuated pathogen establishment and/or expansion within the gut. Interestingly, in terms of *Salmonella* burdens, we discovered two distinct populations of fasted mice. In roughly 40% of mice, fasting severely limited *Salmonella* expansion within the cecum, such that by 24h, either none or only a small number of *Salmonella* (10^4^–10^5^ CFU) could be recovered from their cecal contents and tissues. In contrast, the other 60% of fasted-infected mice carried cecal *S*. Typhimurium burdens similar (10^9^ CFU) to the numbers recovered from fed-infected mice (10^9^) **(Fig 1J)**. Notably, the tissue-protective effects of fasting were seen in all infected mice, as all fasted mice were assessed a pathology score of 0 no matter whether they carried high or low pathogen burdens **(Fig 1G).** Interestingly, no differences were observed between fed and fasted mice regarding *Salmonella* translocating to systemic organs, although overall CFU counts were low and translocation events rare in both groups at 24h post infection (p.i.) **(S1E Fig)**. To examine whether the observed reduction in cecal pathogen burdens could be due to increased antimicrobial peptide (AMP) production in the fasted mice, we measured transcript levels of *RegIII*-*β* and *RegIII*-γ. Expression of both AMPs was significantly decreased in the fasted infected mice, indicating that enhanced AMP production was not causing increased pathogen elimination in the fasted mice **(S1F Fig)**.

We next investigated whether fasting changes the stomach pH as previously reported [21], potentially facilitating the killing of the infectious dose in the upper gastrointestinal tract. However, we found no difference in stomach pH between fed and fasted mice at 24h into their fast (and post antibiotic treatment) **(S1G Fig).** Moreover, at the early timepoint of 12 h p.i. fed and fasted mice showed no difference in pathogen burdens in either their stomachs or small intestines. The cecum proved to be the first site in the GI tract where pathogen burdens significantly differed between fed and fasted mice, with the fasted mice also harboring fewer *Salmonella* in the colon, as well as in their cecal and colonic contents **(S1H Fig).**

To define why so little tissue damage was observed, even in the fasted mice harboring larger numbers of *S*. Typhimurium, we examined *Salmonella’s* spatial localization within the cecum. Similar to our previous studies, immunofluorescence staining showed that in fed mice, *Salmonella* were frequently found within the cecal epithelium, or inside the numerous epithelial cells sloughed into the cecal lumen [22]. In contrast, *Salmonella* in the fasted mice were largely found in the cecal lumen or just above the cecal mucosal surface, with very few intracellular bacteria detected, corresponding to the lack of epithelial cell sloughing seen in these mice **(Fig 1H and 1I)**. This phenotype was observed regardless of whether the fasted mice carried high (≥ CFU 10^6^) or low (CFU 10^3^–10^5^) cecal pathogen burdens **(S1I Fig).**

### Re-feeding restores *S*. Typhimurium invasiveness, but not the host inflammatory response

While these results indicated that the ability of *S*. Typhimurium to invade the intestinal epithelium and cause gastroenteritis was impaired in a fasted host, it was unclear if this effect was irreversible, and whether the lack of an inflammatory response simply reflected the lack of pathogen invasion. We therefore tested the effects of re-feeding an *ad libitum* chow diet for 24h at the end of our 48h fasting experiments (i.e. 48h infection in total). Interestingly, re-feeding led to a dramatic increase in *Salmonella* expansion and invasion into the intestinal epithelial cells (IEC), with large numbers of intracellular bacteria detected by immunofluorescence. Despite this heavy infection, the associated inflammation was still significantly attenuated compared to mice fed throughout the entire 48h of infection. Moreover, cecal epithelial damage and cell sloughing were less pronounced in the re-fed mice **(S2 Fig).** These results suggest that *Salmonella* can rapidly recover their ability to expand and invade IEC following re-feeding, and support the success of the initial antibiotic treatment. In contrast, the attenuated inflammatory response and limited cell sloughing suggest that fasting also directly affects the host response to infection, and that recovery from fasting occurs more quickly for the pathogen, than for the host.

### Fasting does not protect against systemic *S*. Typhimurium infection

**Fig 2:**
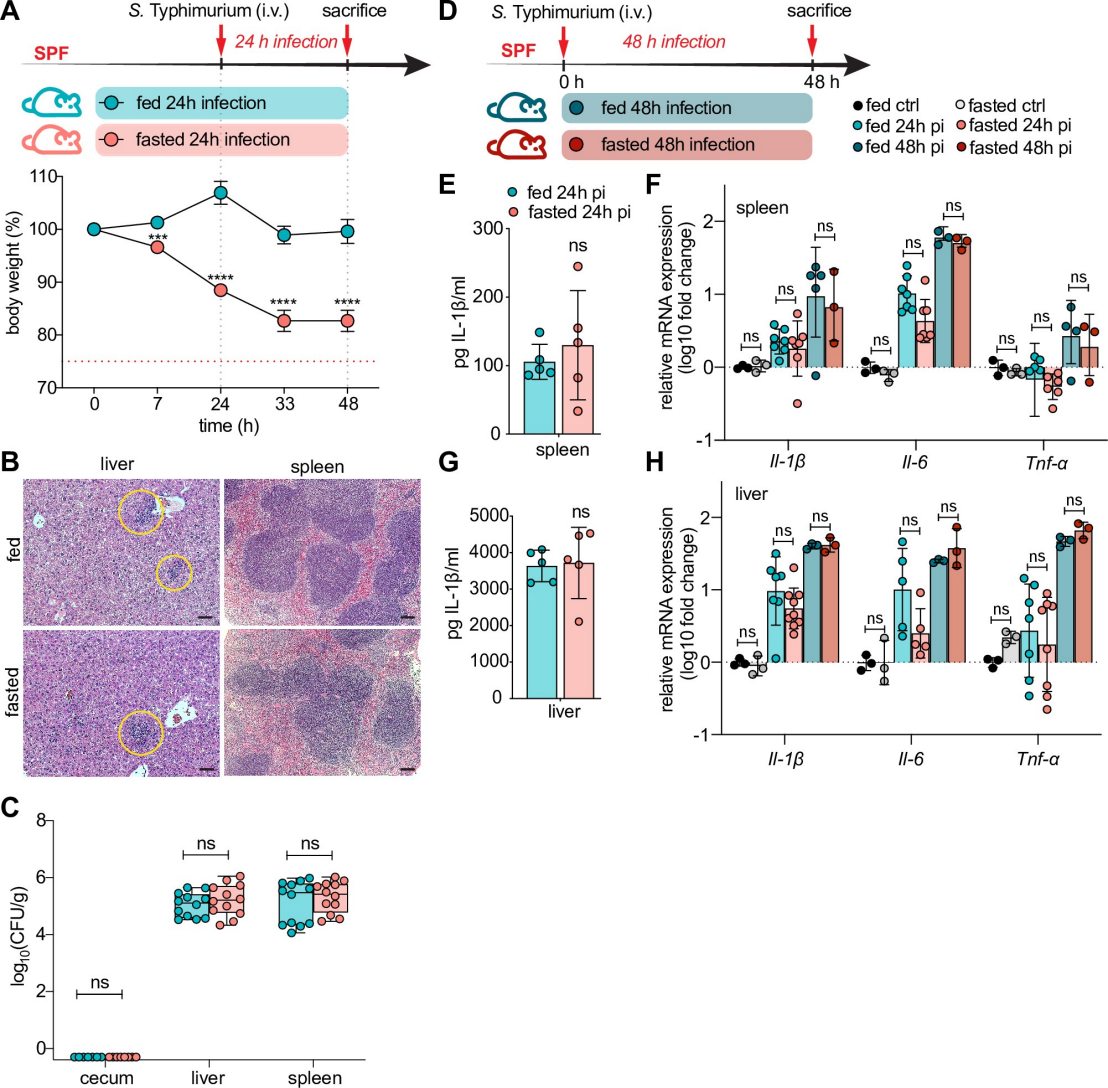
Fasting does not limit systemic *S*. Typhimurium infection. (A, D) Experimental timeline of infection and fasting regimen (applicable to all data shown in this figure) and concurrent body weight loss. SPF C57BL/6 mice were *i*.*v*. (tail vein) injected with ~ 5 × 10^4^ CFU *S*. Typhimurium and sacrificed at 24h p.i. (A) (n = 12) or 48h p.i. (D) (n = 6) (B) Representative H&E-stained liver and spleen sections of mice *i*.*v*. infected with *S*. Typhimurium at 24h p.i. ± 48h of fasting. Yellow circles indicate accumulation of granulocytes. Scale bar 50 μm. (C) *S*. Typhimurium CFU per g tissue 24h p.i. (see Fig 2A for experimental timeline). (E, G) IL-1β protein levels measured by ELISA in spleen and liver lysates of mice *i*.*v*. infected with *S*. Typhimurium at 24h p.i. ± 48 h of fasting. (F, H) qPCR analysis of inflammatory genes in mouse spleen (F) and liver (H) expressed as fold change over fed ctrl. Fed ctrl and fast ctrl refer to uninfected *ad libitum* fed or 48h fasted C57BL/6 SPF mice. All data shown repeated in 3 independent experiments. For (C), (F) and (H) data from multiple independent experiments were pooled. **** p < 0.0001, *** p < 0.001, ns = not significant. Significance levels calculated by unpaired Student’s t test (E, G), multiple t-test (A) and Mann-Whitney-Wilcoxon test (C) with Holm-Sidak correction and 2-way ANOVA with Tukey post-test (F, H). Error bars shown as ± SD, except (C) which is shown as box plot with min-max whiskers.

In light of the dramatic protective effects on cecitis observed during fasting, we examined whether this protection was gut-specific, or if fasting could also protect against systemic spread of *S*. Typhimurium following intravenous infection. C57BL/6 mice were fed or fasted for 24h, and then intravenously infected with 5 x 10^4^ CFU *S*. Typhimurium and respective groups subsequently fed or fasted throughout the following 24h **(Fig 2A)**. Spleen and liver tissues were collected 24h p.i. to analyze *S*. Typhimurium numbers. Interestingly, fasting had no overt effect on the pathogen burdens recovered from these tissues, nor on the histopathology in these organs **(Fig 2B and 2C)**. Since no overt pathology and only a mild inflammatory response could be seen even in the fed mice after 24h of infection **(Fig 2E–2H)**, we added another group of mice that underwent a 48h systemic infection, and thus developed a stronger inflammatory response. Nevertheless, gene transcription of major pro-inflammatory cytokines did not differ between the fed and fasted groups **(Fig 2D, 2F and 2H).** Thus, fasting appears to selectively affect *S*. Typhimurium’s pathogenesis within the GI tract.

### Fasting alters the gut microbiome but not the efficacy of the streptomycin treatment

**Fig 3:**
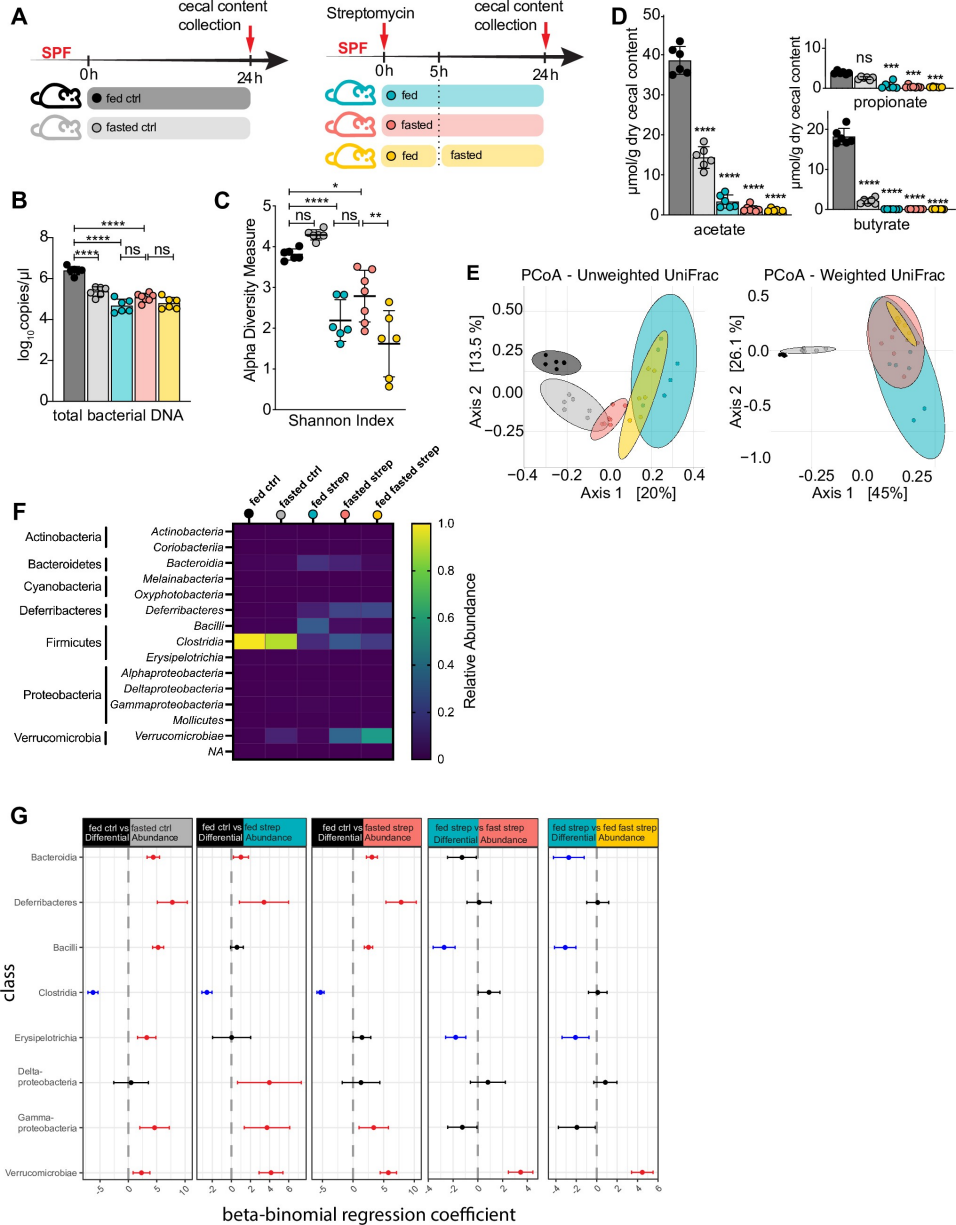
Fasting alters the gut microbiome but does not affect the efficacy of streptomycin. (A) Experimental timeline of streptomycin treatment and fasting regimen (applicable to all data shown in this figure). SPF C57BL/6 mice were orally gavaged with 20 mg of streptomycin and fed or fasted for the next 24h. An additional group was given streptomycin, fed for another 5h and fasted for the following 19h. Fed ctrl and fasted ctrl refer to non-streptomycin treated *ad libitum* fed or 24h fasted C57BL/6 SPF mice. For all groups, mice were euthanized after 24h and cecal contents collected. (B) ddPCR quantification of eubacterial DNA copies in DNA extracted from cecal contents of C57BL/6 SPF mice. (C, E-G) 16S rRNA analysis of cecal microbiome of C57BL/6 SPF mice. (C) Microbial biodiversity measured by Shannon Index. (D) Short chain fatty acids (acetate, butyrate and propionate) were measured in cecal contents and normalized to dry weight. Significance level depicts each group compared to fed control. (E) Principal Coordinates Analysis of unweighted and weighted UniFrac distances visualizing β-diversity. Ellipses represent 95% confidence intervals. (F) Relative abundance of bacterial class in each group. (G) Pair-wise differential abundance at class level using beta-binomial regression. Red bars indicate significant positive changes and blue bars indicate significant negative changes while black bars indicate no difference in relative abundance in the respective comparison. **** p < 0.0001, *** p < 0.001, ** p < 0.01, * p < 0.05, ns = not significant. Significance levels calculated by 1-way (B, C) and 2-way ANOVA (D) with Tukey post-test. Error bars shown as ± SD. Related to S3 Fig.

The presence of commensal microbes significantly impairs *S*. Typhimurium’s ability to infect the healthy mouse intestine. This “colonization resistance” is initially overcome through oral streptomycin treatment prior to infection [16,23]. Considering the effects of fasting on *S*. Typhimurium pathogenesis in the GI tract, we decided to further confirm whether fasting affected the efficacy of the antibiotic streptomycin, thus impairing infection. We focused on the status of the intestinal microbial community just prior to infection (24h after streptomycin treatment and 24h into the fast). We therefore analyzed microbiome changes using 16S rRNA analysis of cecal contents in the two different treatment and respective control groups, as well as a group (termed fed-fasted) that started the fast 5h after streptomycin treatment, providing more time for streptomycin to work under fed conditions **(Fig 3A)**. These fed-fasted mice thus underwent a shorter fast, but were still found to be protected from *S*. Typhimurium-induced cecitis **(S3A–S3D Fig)**. Details on the diet, animal sourcing, housing and other factors that may impact the gut microbiome are outlined in the Materials and Methods section.

We quantified commensal bacteria in the cecal contents of fed and fasted mice, both with and without streptomycin treatment. As expected, antibiotic treatment significantly reduced commensal numbers in the ceca, but in both fed and fasted mice. This indicates that fasting did not disrupt streptomycin’s ability to deplete a portion of the gut microbiota **(Fig 3B).** This decrease in commensal abundance was accompanied by a reduction in bacterial alpha diversity in all streptomycin treated groups as measured by their Shannon Index **(Fig 3C)**.

Previous studies have shown that streptomycin pre-treatment leads to depletion of butyrate-producing bacteria, specifically those in the phylogenetic class of Clostridia, which is key to the loss of colonization resistance seen in this model [24]. We therefore examined the relative abundance of Clostridia as well as butyrate (plus acetate and propionate) levels in cecal contents and again found similar dramatic reductions in fed and fasted (and fed-fasted), streptomycin treated mice **(Figs 3D, 3F and S3F).** This confirms that fasting does not prevent the typical depletion of butyrate producing microbes required in this model. Notably, while fasting on its own, independent of streptomycin treatment, greatly diminished the number of commensals, bacterial alpha diversity was not affected **(Fig 3B and 3C)**. Similarly, a sharp drop in butyrate levels was also measured in the fasted control group, yet the relative abundance of Clostridia in this group did not decrease to the same extent as observed in the streptomycin-treated mice. This drop in butyrate is likely explained by the lack of fermentable substrate (fiber) in the fasted group, thus preventing butyrate production **(Figs 3D, 3F and S3F)**.

Next, we sought to identify a distinct microbial signature associated with the fasting-mediated protection from *Salmonella-*induced gastroenteritis. Principle coordinate analysis revealed clustering patterns for each group measured with either weighted or unweighted UniFrac. Weighted UniFrac analysis, which considers the relative abundance of the bacterial community members, revealed that all streptomycin-treated groups cluster together away from both control groups, highlighting that the antibiotic treatment led to a shift in microbial abundance (i.e. likely reflecting the loss of butyrate producers) regardless of nutrient status. Unweighted UniFrac analysis, which accounts for the presence/absence of microbial community members and thus emphasizes rare lineages, showed individual clusters for each group indicating that community composition was affected by both nutrient status and antibiotic treatment. Thus, antibiotic exposure as well as fasting create unique environments that give rise to specific, low-abundance community members **(Fig 3E).**

To further specify the distinct microbial signature each treatment introduced, we applied differential abundance analysis at the class level using a beta-binomial regression model. This revealed taxa that were significantly differentially abundant between groups **(Fig 3G).** Interestingly, the most significant change between fed and fasted, streptomycin-treated cecal microbiomes was the differential relative abundance of Verrucomicrobiae to which the genus *Akkermansia* belongs **(Figs 3G, S3E and S3H).** Furthermore, the classes of Bacilli and Erysipelotrichia were significantly less abundant in the fasted streptomycin-treated mice than in their fed counterparts. We also examined the differential abundance of *Enterobacteriacae* as it has been shown that members of this family can confer colonization resistance to mice orally infected with *Salmonella*. However, this group was only significantly increased in the fed antibiotic-treated group, ruling out that the fasted mice were protected due to an increase in *Enterobacteriacae*-mediated colonization resistance **(S3G Fig)** [25].

The presence of a distinct microbial profile associated with the fasted group along with the drop in pathogen load specifically in the cecum but not in the stomach or small intestine of fasted mice **(S1H Fig)** led us to next test whether the intestinal microbiota is required for the protective effects of fasting in preventing *S*. Typhimurium-induced gastroenteritis.

### Fasting protects germfree mice from gastroenteritis but not from epithelial cell invasion

**Fig 4:**
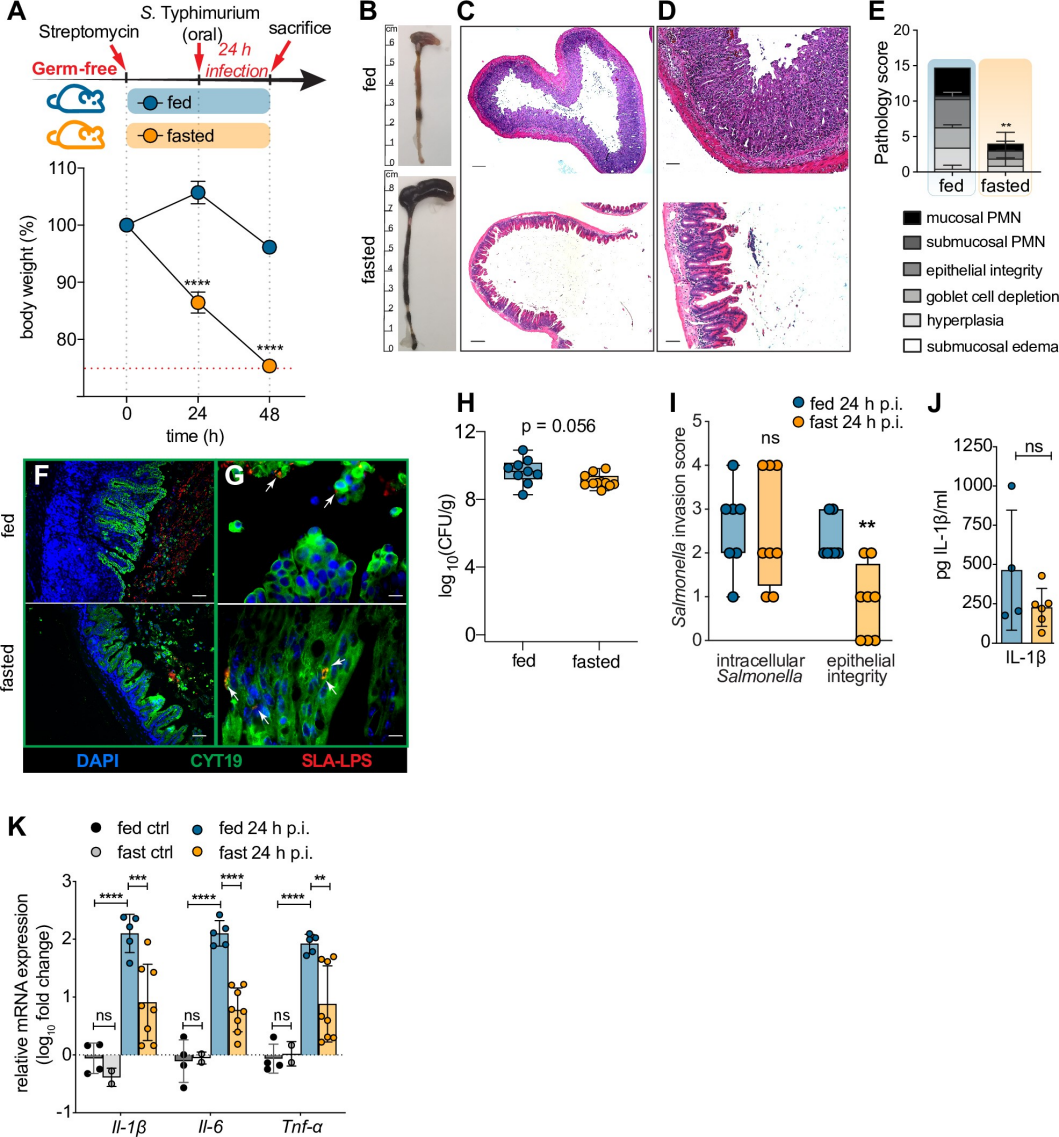
Fasting protects germfree mice from severe gastroenteritis but not from epithelial cell invasion. (A) Experimental timeline of infection and fasting regimen (applicable to all data shown in this figure) and concurrent body weight loss. Streptomycin-pretreated GF C57BL/6 mice were orally gavaged with ~ 2.5 × 10^6^ CFU S. Typhimurium and euthanized 24h p.i. (n = 7). (B) Representative macroscopic images of GF mouse cecum and colon pathology 24h p.i. ± 48h of fasting. (C-D) Representative H&E-stained cecal sections of GF mice at 24h p.i.± 48h of fasting. Scale bar 200 μm (C), 50 μm (D). (E) Histopathological analysis of GF mouse cecal H&E tissue sections (see Materials and Methods for scoring criteria). Agreement among raters ensured by Kendall’s coefficient of concordance Wt = 0.737 (n ≥ 7). (F-G) Representative immunofluorescence staining of *S*. Typhimurium and IEC on GF mouse cecal sections at 24h p.i. ± 48h of fasting. Sections were stained using DAPI to detect DNA (blue), anti-*Salmonella*-LPS (SLA, red) to visualize *S*. Typhimurium and anti-cytokeratin 19 (CYT19, green) to stain epithelial cells. White arrows indicate intracellular *Salmonella*. Scale bar 50 μm (F), 10 μm (G). (H) *S*. Typhimurium CFU per g cecal tissue and stool (combined) 24h p.i. ± 48h of fasting in GF mice. (I) Invasion score quantifying *S*. Typhimurium presence in IEC by analyzing immunofluorescently stained GF mouse cecal sections at 24h p.i. ± 48h of fasting (shown in F-G) (see Materials and Methods for scoring criteria). Agreement among raters ensured by Kendall’s coefficient of concordance Wt = 0.926 (n ≥ 7). (J) IL-1β protein levels measured by ELISA in whole cecum lysates of GF mice infected with *S*. Typhimurium at 24h p.i. ± 48h of fasting. (K) qPCR analysis of main inflammatory genes in GF mouse cecum expressed as fold change over fed ctrl. Fed ctrl and fast ctrl refer to uninfected *ad libitum* fed or 48h fasted C57BL/6 GF mice. All data shown pooled from multiple independent experiments. **** p < 0.0001, *** p < 0.001, ** p < 0.01, * p < 0.05, ns = not significant. Significance levels calculated by unpaired Student’s t test (E, J), multiple t-test (A), Mann-Whitney-Wilcoxon test (H, I) with Holm-Sidak correction, and 2-way ANOVA with Tukey post-test (K). Error bars shown as ± SD (A, E, J, K); box plot with min-max whiskers (H, I). Related to S4 Fig.

To address whether it was commensal microbes that prevented *Salmonella* from infecting the intestinal mucosa of fasted mice, we obtained germfree (GF) C57BL/6 mice, and tested their responses to *S*. Typhimurium under fed and fasted conditions. Mice underwent the same procedures outlined for specific-pathogen free (SPF) mice, including pretreatment with streptomycin **(Fig 4A),** however streptomycin treatment itself had no overt effect on cecal histopathology **(S4A and S4B Fig)**. As shown in **Fig 4B–4D**, fed GF mice proved highly susceptible to *S*. Typhimurium infection, carrying heavy intestinal pathogen burdens (10^9^) and suffering severe gastroenteritis including widespread epithelial cell invasion and sloughing.

Notably, fasting still showed protective effects in GF mice, albeit less pronounced than in SPF mice. Fasted infected GF mice showed increased inflammatory markers compared to uninfected control GF mice **(Fig 4J and 4K)** along with evidence of small numbers of epithelial cells sloughing into the cecal lumen, but pathology scores were still dramatically reduced compared to fed infected GF mice **(Fig 4B–4E)**. Similiar to SPF mice, fasting also reduced AMP expression in infected GF mice **(S4D Fig**). Interestingly, all fasted GF mice harbored large pathogen burdens, comparable to those seen in fed GF mice **(Fig 4H).** Also comparable to SPF mice, no difference could be observed between fed and fasted GF mice in their systemic pathogen burdens, indicative of *Salmonella* translocation **(S4C Fig)**. Strikingly, quantification of intracellular *Salmonella* by immunofluorescence staining revealed that in contrast to their fasted (but SPF) counterparts, the cecal epithelium of GF fasted mice was heavily infected, with numerous *S*. Typhimurium identified within their IEC **(Fig 4F, 4G and 4I)**. Even so, the typical inflammation and IEC sloughing seen in infected fed GF mice was still significantly attenuated in the fasted group **(Fig 4J and 4K)**.

### Fasting suppresses mucosal NF-κB expression and downstream inflammatory mediators

**Fig 5:**
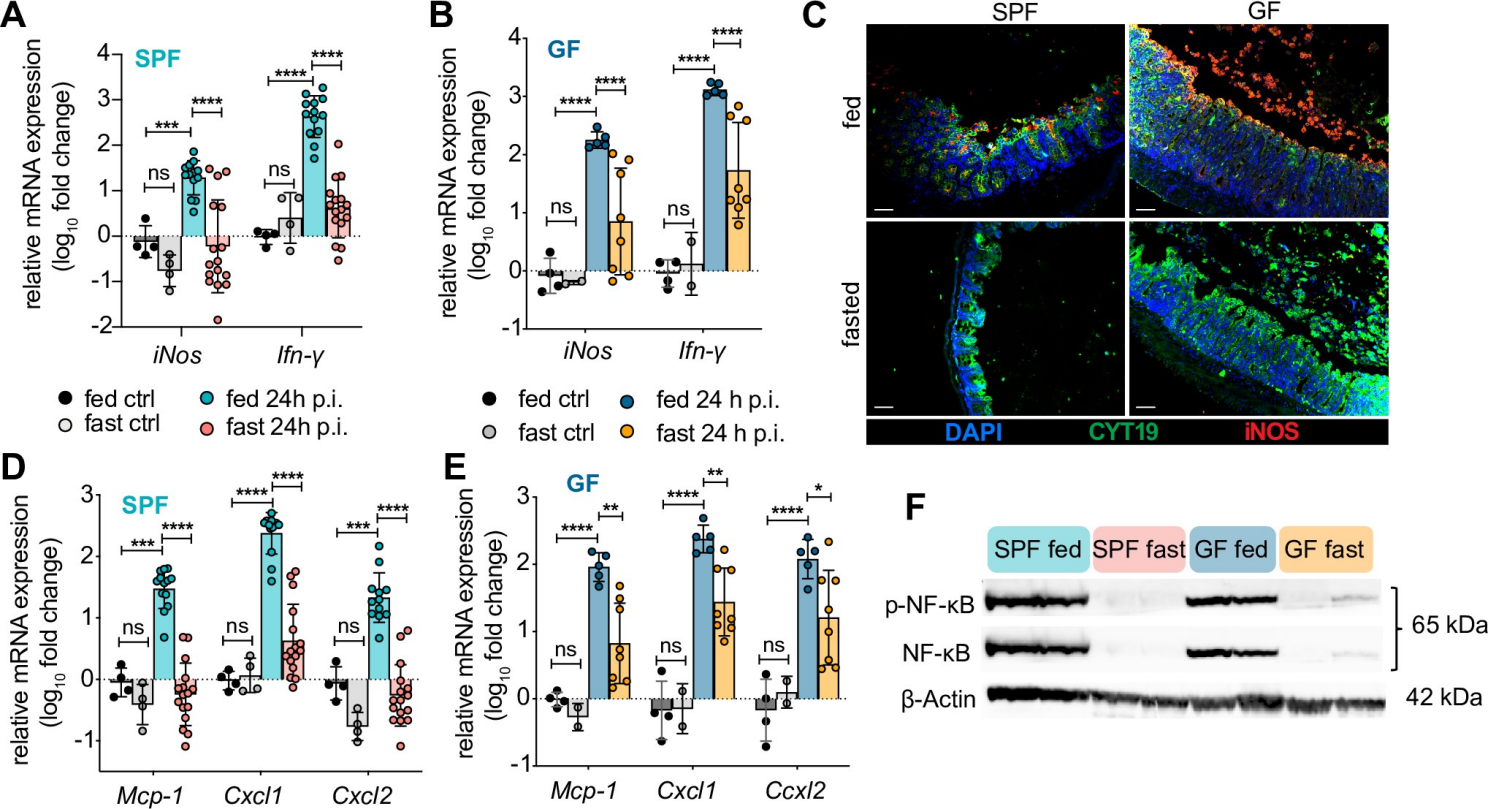
Fasting suppresses mucosal NF-κB expression and downstream inflammatory mediators. (A-B, D-E) qPCR analysis of *iNos* and *Ifn-γ* mRNA expression in SPF (A) and GF (B) and different chemoattractants in SPF (D) and GF (E) *S*. Typhimurium-infected mouse ceca, expressed as fold change over fed ctrl. Fed ctrl and fast ctrl refer to uninfected *ad libitum* fed or 48h fasted C57BL/6 GF or SPF mice. (C) Representative immunofluorescence staining of inducible nitric oxide synthase (iNOS) and IEC on SPF and GF cecal sections at 24h p.i. with *S*. Typhimurium ± 48h of fasting, n = 6. Sections were stained using DAPI to detect DNA (blue), anti-iNOS (iNOS, red) to visualize iNOS expression and anti-cytokeratin 19 (CYT19, green) to stain IEC. Scale bar 50 μm. (F) Representative immunoblot detecting phosphorylated and total NF-κB p65/RelA protein in whole cecal tissue lysates of SPF and GF mice at 24h p.i. with S. Typhimurium ± 48h of fasting. Lanes represent data from individual mice, n ≥ 5. Equal loading confirmed with β-actin as well as total protein stain of membrane (see S5 Fig). All data shown repeated in at least 3 independent experiments. For (A, B, D, E) data from multiple independent experiments were pooled. **** p < 0.0001, *** p < 0.001, ** p < 0.01, * p < 0.05, ns = not significant. Significance levels calculated by two-way ANOVA with Tukey post test. Error bars shown as ± SD. Related to S5 Fig.

The attenuation of the characteristic cecal inflammation and IEC sloughing seen in fasted infected GF mice, suggests fasting also modulates the host immune response to infection. Along with the attenuated expression of *Il-1β*, *Il-6* and *Tnf-α* in fasted infected SPF and GF mice **(Figs 1K, 1M, 4J and 4K)** we observed attenuated expression of another key pro-inflammatory cytokine—*Ifn-γ* **(Fig 5A and 5B)**. Among its actions, this cytokine potently activates inducible nitric oxide synthase (iNOS) expression by different cell types, including IEC [26,27]. We thus examined cecal iNOS expression to better characterize the inflammatory response by IEC in the fasted infected mice. Indeed, *iNos* mRNA expression followed the same pattern as *Ifn-γ* expression **(Fig 5A and 5B).** Moreover, immunostaining confirmed that iNOS expression was readily observed in the cecal epithelial cells of fed-infected SPF and GF mice. In contrast, iNOS expression was lacking in most fasted SPF mice and almost completely absent in GF fasted mice, even when assessing those fasted GF mice that showed moderate cecal pathology **(Fig 5C)**. Additional transcriptional analysis of several important chemokines (*Mcp-1*, *Cxcl1*, *Cxcl2*) followed a similar pattern: Strongly induced in fed SPF and GF mice, significantly blunted in fasted SPF mice, modestly, but still significantly blunted in fasted GF mice **(Fig 5D and 5E).**

Common to all these inflammatory mediators and chemokines is their upstream transcriptional control through NF-κB [28]. The transcription factor NF-κB is a central regulator by which IEC and immune cells mediate downstream innate signaling in response to bacterial infections [29,30]. Moreover, fasting has been shown in other disease models to suppress several inflammatory pathways, including NF-κB [31]. To clarify if fasting inhibited NF-κB in our infection model, we collected cecal tissues from fed and fasted–infected SPF and GF mice. We performed Western blots for NF-κB (p65/RelA subunit) and its respective phosphorylated state. As expected, NF-κB was strongly activated in the cecal tissues of fed mice, yet was absent in the tissues of fasted SPF and GF mice, despite the heavy presence of *Salmonella* within the cecal mucosa of GF mice **(Fig 5F)**. Thus, fasting reduces inflammatory responses to *S*. Typhimurium within the intestinal mucosa via suppression of NF-κB expression, thereby limiting the subsequent gastroenteritis.

### *S*. Typhimurium is unable to effectively induce SPI-1 in the intestines of fasted SPF mice but retains this ability in GF fasted mice

While the above results focused on the role of fasting in modulating host responses during *Salmonella* infection, we questioned whether fasting would also impact the expression of *Salmonella* virulence genes. To test this, we examined the expression of SPI-1 virulence factors, since they are required for *Salmonella* to infect host IEC and trigger gastroenteritis. SPF mice were infected with *S*. Typhimurium harboring plasmid pCS26-*invF* containing a *luxCDABE* transcriptional fusion under the control of the *invF* promoter (*invF-lux*)–the gene encoding a *Salmonella* transcriptional regulator required for expression of SPI-1 effector proteins–as a reporter of the SPI-1 HilD-HilA-InvF regulatory cascade [32].

**Fig 6:**
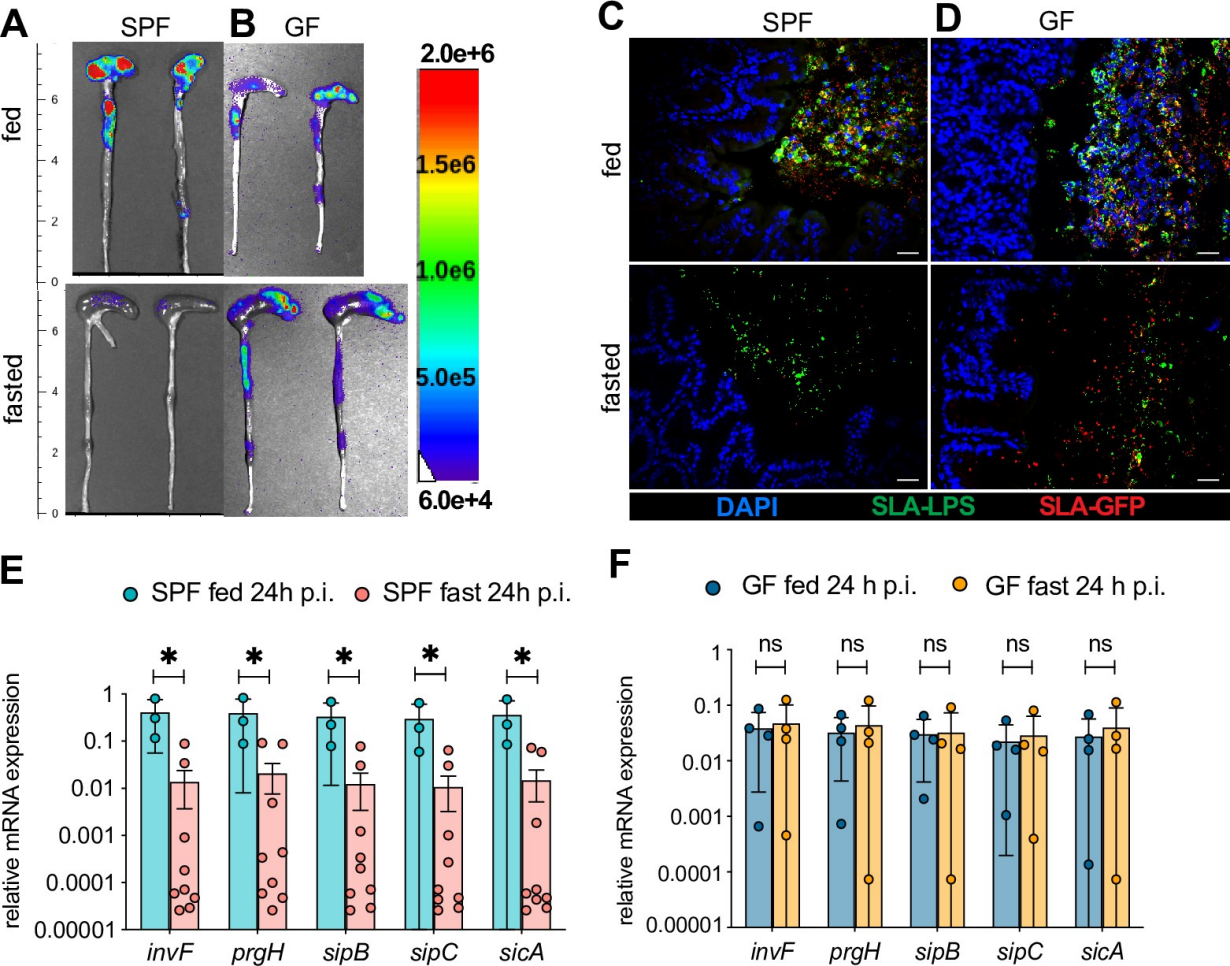
*S*. Typhimurium is unable to induce SPI-1 in the intestines of fasted SPF mice but retains this ability in GF fasted mice. (A, B) Representative macroscopic *ex vivo* images of SPF and GF mouse cecum and colon showing magnitude of bioluminescence signal corresponding to expression of the *Salmonella* SPI-1 *invF-luxCDABE* transcriptional fusion. Streptomycin-pretreated mice were orally infected with *S*. Typhimurium carrying the *invF-luxCDABE* transcriptional fusion in plasmid pCS26-*invF*, at a dose of ~ 2.5 × 10^8^ CFU. Mice were euthanized and intestines imaged at 15h p.i. ± 39h of fasting. Bioluminescence signals were measured in logarithmic units of light (photons/s/cm^2^/sr). (C, D) Representative immunofluorescence staining on paraffin embedded SPF and GF mouse cecal sections at 15h p.i. ± 39h of fasting. Streptomycin-pretreated mice were orally infected with ~ 2.5 × 10^8^ CFU *S*. Typhimurium carrying a plasmid expressing destabilized GFP [GFP(LVA)] under the control of the promoter of the gene encoding the SPI-1 T3SS needle complex protein *prgH*. Sections were stained using DAPI to detect DNA (blue), anti-Salmonella-LPS (SLA-LPS, green) to visualize total number of *S*. Typhimurium and anti-GFP to detect *prgH*-positive *Salmonella* (SLA-GFP, red). Scale bar 50 μm. (E, F) Expression of several SPI-1 virulence genes normalized to *Salmonella rrsA* (16S) gene expression in cecal content of *S*. Typhimurium infected SPF (E) or GF (F) mice at 24h p.i. ± 48h of fasting, as quantified by ddPCR. For (A, B, D, E) data from multiple independent experiments were pooled. * p < 0.05, ns = not significant. Significance levels calculated by multiple t-test with Holm-Sidak post test. Error bars shown as ± SD. Related to S6 Fig.

An infection time course revealed that SPI-1 driven luciferase expression was strongly induced in the intestines of *S*. Typhimurium *invF-lux*-infected fed SPF mice, with a maximal signal seen at 15h p.i. (see **Fig 6A).** Since SPI-1 induction was not synchronized among the whole population of *S*. Typhimurium, the strong luciferase signal detected from the infected tissues represented a subset of the bacteria, and is not necessarily, directly proportional to the recovered CFU counts. Thus, we only compared and quantified sets of tissues where fasted mice had similar cecal pathogen burdens as fed mice. Despite the presence of numerous *Salmonella*, a 10-fold reduction in SPI-1 signal was observed in the fasted mice at 15h p.i. (**Figs 6A and S6B**). To exclude the possibility that the reduction in luminescence signal (which is oxygen-dependent), in the fasted mice was merely reflective of reduced tissue oxygenation due to the absence of inflammation in the fasted intestines, we infected fed and fasted mice with *Salmonella* constitutively expressing the *Photorhabdus luminescens* lux operon on the chromosome. As seen in **S6A Fig**, the luminescence signal was similar between fed and fasted mice. Furthermore, when quantifying expression of the *Salmonella* SPI-1 *invF-luxCDABE* in washed ceca or isolated stool–a process which allows for ample oxygenation–no increase in luciferase signal from the fasted tissues could be detected **(S6D and S6E Fig)**. These data suggest that fasting suppresses the induction of *Salmonella*’s SPI-1 program typically seen in the intestines of fed mice and that the observed reduction in luciferase signal in fasted mice was not due to decreased oxygen availability.

To further evaluate the effects of fasting on SPI-1 expression, we also infected mice with a *Salmonella* strain expressing destabilized GFP[LVA] under the control of the *prgH* (another SPI-1 gene) promoter (*prgH-*GFP) [33]. Tissues from *prgH*-GFP infected mice were subsequently immunostained for GFP and *S*. Typhimurium LPS. The ceca of infected fed mice showed widespread GFP staining (red) co-localizing with almost all of the *Salmonella* (detected with anti-LPS antibodies, green) near the mucosal surface (**Fig 6C**). While it has been previously described that not all *Salmonella* infecting host cells express SPI-1, this particularily involves the spatial segregation of SPI-1 expression [34]. Specifically, *Salmonella* populations located close to or in contact with the epithelium or sloughed IEC (as shown here in fed mice) preferentially express SPI-1 [35]. In contrast, in the fasted mice, fewer than 5% of the *Salmonella* near the mucosal surface were *prgH*-positive **(Fig 6C)**.

Since both methods are only semi-quantitative measurements of SPI-1 transcriptional activity, we also quantified mRNA transcription of several SPI-1 genes in the cecal contents of fed and fasted infected SPF mice. Indeed, we observed a 10-10000-fold reduction in SPI-1 gene transcription in the fasted SPF mice as compared to their fed counterparts **(Fig 6E).**

Taken together, these data indicate that the lack of SPI-1 induction by *S*. Typhimurium in the fasted SPF mice was likely responsible for their inability to infect the intestinal epithelium. Since *Salmonella* did infect the cecal epithelium of GF mice, we examined whether fasting had any effect on SPI-1 induction by *Salmonella* when infecting GF mice. Correspondingly, fed and fasted GF mice were infected with the luciferase and GFP expressing *Salmonella* strains (*invF-lux* and *prgH*-GFP) and the mRNA of several SPI-1 genes was measured in the cecal contents of fed and fasted mice. As expected, SPI-1 was strongly induced in *Salmonella* found in the intestines of fed GF mice, but it was also induced in infected-fasted GF mice **(Figs 6B, 6D, 6F and S6C)**.

These data indicate that in fasting SPF mice, it is the commensal microbes that limit *Salmonella*’s ability to invade the intestinal epithelium, as a form of colonization resistance.

### The ability of fasting to suppress enteric bacterial infections is not limited to *S*. Typhimurium

While fasting exerted a dramatic suppressive effect on the virulence program used by *S*. Typhimurium to infect the mammalian GI tract, it remained unclear whether fasting would suppress the ability of other enteric bacterial pathogens to cause gastroenteritis. To test this, we implemented a similar fasting regimen in a mouse model of *Campylobacter jejuni*-induced gastroenteritis **(S7 Fig)**. In this model, *Sigirr* ^-/-^ mice (deficient in single immunoglobulin and toll-interleukin 1 receptor domain (SIGIRR)), were orally gavaged with vancomycin followed 5h later by oral infection with ~ 1 x 10^7^ CFU *C*. *jejuni* [36]. Mimicking the *Salmonella* fasting protocol, *Sigirr* ^-/-^ mice were either fed or fasted for the 5h post-antibiotic treatment and throughout the following 43h infection period (48h fast in total) (**S7A–S7D Fig**). Signs of cecitis usually take 72h to develop in this model, so tissue pathology was not assessed. Even so, we were still able to evaluate the expansion of *Camplyobacter* within the cecum and stool at 43h p.i. **(S7E–S7G Fig**). Interestingly, fasting significantly decreased *C*. *jejuni* burdens in the cecal tissues and luminal contents of these mice by ~1000-fold. These findings indicate that a fasted host not only poses a challenging environment for *Salmonella*, but that fasting can inhibit the expansion of another enteric pathogen, *C*.*jejuni* as well. As such, the protective effects of fasting may be generalizable to a broad range of enteric bacterial pathogens.

## Discussion

Infection-induced fasting is a conserved behavior that likely evolved as part of a generalized host response to infection. Moreover, fasting-based therapies are gaining recognition and acceptance for their ability to suppress maladaptive immune activation. Since it remains controversial whether anorexic behavior/fasting protects a host, or instead increases their susceptibility to pathogens, mice were fasted before and during an oral *Salmonella* infection, with assessments made of pathogen virulence, host immunity and the resident gut microbiome. Here we show that prolonged fasting provides dramatic benefits to the host during the course of an enteric infection. Along with eliciting microbiota-dependent changes in the intestinal environment that prevent *Salmonella* from invading IEC, it also suppressed the typical inflammatory responses that *Salmonella* and other enteric pathogens both trigger and exploit [37]. As a result, even though *Salmonella* retained their ability to invade the intestinal epithelium in GF mice lacking a gut microbiota, they were unable to trigger the severe inflammation and tissue damage typically seen in this model. These findings highlight the importance of food intake in regulating the complex relationship that exists between intestinal microbes and their hosts.

Infection-induced loss of appetite occurs once the pathogen is sensed by the host. For Gram-negative bacterial infections, this response is elicited through a lipopolysaccharide (LPS)-induced-peripheral IL-1β release which in turn stimulates the release of brain-endogenous cytokines that act on hypothalamic appetite control. Depending on the route and severity of the infection as well as the type of pathogen, the onset of the anorexic response can be a few hours (3-6h) to several days following infection and can last up to a week thereafter [7,38]. Since anorexia is already a feature of most enteric infections, we implemented fasting prior to infection to interrogate its impact on the virulence of pathogens entering a fasted environment. Moreover, we wanted to assess how fasting affected the inflammatory response to *Salmonella*, as this is important with respect to therapeutic fasting and how it may impair host defense systems.

During enteric infection the first barrier encountered by *Salmonella* is the resident commensal microbial community. In the *Salmonella* gastroenteritis model, it is necessary to deplete specific commensal microbes to overcome colonization resistance, and allow *Salmonella* to expand and establish itself in the host intestine [16]. Studies by Rivera-Chávez and colleagues have shown that streptomycin pretreatment, as well as specific *Salmonella* virulence strategies drive depletion of *Clostridia* species, which in turn leads to butyrate depletion in the gut and increased oxygenation of colonocytes. *Salmonella* is able to expand in this aerobic environment and cause gastroenteritis [24]. We also observed streptomycin-induced depletion of *Clostridia* as well as butyrate and propionate depletion in cecal contents in both fed and fasted mice. In concert with our finding that previously infected fasted mice developed gastroenteritis upon refeeding, these data confirm that fasting did not hamper the efficacy of the streptomycin treatment. Interestingly, butyrate, as well as propionate, are known to down-regulate SPI-1 gene expression [39]. Thus, differences in butyrate/propionate levels are unlikely to be the cause of the decreased bacterial invasion seen in the fasted state. Acetate, in contrast a known activator of SPI-1 gene expression [40], was also not significantly different in the cecal contents of fed and fasted mice, ruling out this regulatory mechanism as well.

The inability of *Salmonella* to effectively induce its SPI-1-regulated virulence program in fasted SPF mice must therefore reflect other mechanisms. Since *Salmonella* were able to express SPI-1 and invade the intestinal epithelium of GF mice, regardless of their fed or fasted state, this argues that competition for resources with commensal microbes might be at play. Studies have shown that expression of the SPI-1 type III secretion system (T3SS) can be modulated by nutrient availability [41]. Specifically, *Salmonella* cultured in minimal media show reduced expression of SPI-1 effectors *in vitro* [42]. While we infected mice with *Salmonella* grown in complete LB medium, it was likely the *in vivo* competition for nutrients with commensals that led to the suppression of SPI-1 in fasted SPF mice. If so, fasting could potentially inhibit other invading bacterial pathogens from colonizing the GI tract, and correspondingly, we found fasting also protected against *C*. *jejuni* infection.

That nutrient competition with commensals likely plays a role in the fasted-infected mice is also highlighted by our observations at 12h p.i., seeing that *Salmonella* pathogen burdens in fed and fasted mice were similar in the upper gastrointestinal tract (stomach, ileum) but were significantly decreased in the fasted mice once *Salmonella* reached the cecum. Notably, the cecum is also the first site where gavaged *Salmonella* would encounter a large commensal bacterial community, and thus represents the first place in the GI tract where the pathogen would face significant competition for nutrients.

While such colonization resistance likely reflects the actions of many different species of gut microbes, 16S rRNA analysis revealed the genus *Akkermansia* was more abundant across all fasted groups. This microbe consumes intestinal mucus and increases in abundance in response to fasting in hamsters, pythons and humans [43–46]. This might reflect a conserved response by the microbiome to directly “feed off the host” in times of starvation. Notably, *Akkermansia* are reduced in IBD patients and in some cases, associated with a healthy gut microbiome [47]. While it is possible that the increase in *Akkermansia* could play a role in suppressing *Salmonella* invasion of epithelial cells, another study found that an increase in *Akkermansia* in fact rendered mice more susceptible to *Salmonella* and *Citrobacter rodentium* infections [48,49].

In contrast, the relative abundance of Bacilli and Erysipelotrichia, both belonging to the Firmicutes phylum was found to be significantly decreased in the fasted mice as compared to the fed antibiotic treated mice. While it is not clear if this decrease has an impact on pathogen expansion in our model, a recent study reported a diet-specific decrease in the relative abundance of *Erysipelotrichaceae*, a prominent class member of Erysipelotrichia, following streptomycin treatment. In this case, no difference in pathogen burden was observed between diets following *Salmonella* infection, indicating that our observed decrease in *Erysipelotrichaceae* may not be contributing to the increased colonization resistance seen in fasted mice [50]. On the other hand, a study examining the effect of *Salmonella* infection on the microbiome without streptomycin-pretreatment found that infected mice carrying high relative abundance of *Salmonella*–termed “high responders”—were co-enriched with Bacilli, while “low responders” had significantly lower abundance of Bacilli. However, this change in Bacilli abundance was not a marker of inflammation itself as it was exclusively observed in the context of *Salmonella-*induced inflammation and did not occur with dextran sodium sulfate (DSS)-induced inflammation. As such, the relatively low abundance of Bacilli we observed in the fasted group post streptomycin treatment might be linked to the subsequent lower burdens of *Salmonella* in these mice [51].

Future studies using higher resolution microbiome sequencing in conjunction with germfree mouse colonization experiments should tease apart whether the presence or absence of one or multiple commensal species is sufficient to confer the fasting-induced protection or whether it might be the metabolic competition for nutrients between commensals and pathogens that is responsible for the effect seen, independent of the intestinal microbial composition.

Notably, we saw no protective effect of fasting when *Salmonella* was injected *i*.*v*. and spread to the liver and spleen. This emphasizes the key role played by the gut microbiome in mediating the protective effects of fasting. Interestingly, when Rao *et al*. showed that *Salmonella* infection-induced anorexia was detrimental, they performed oral infections in the absence of streptomycin treatment. In that model, *Salmonella* does not cause gastroenteritis, but instead the infection progresses quickly to systemic sites (liver and spleen), mimicking typhoid fever. In their study, exaggerated host anorexia, due to loss of the *Salmonella* effector SlrP, proved harmful and led to increased mortality in the susceptible mouse strain used for infection. This likely reflects the ability of SlrP to suppress the release of IL-1β by infected host cells. It was found that WT *Salmonella* (expressing SlrP) inhibited host anorexia, potentially to promote its own survival and transmission at the cost of its virulence [7]. It remains unclear whether loss of SlrP would also alter the course of *Salmonella-*induced gastroenteritis, and concurrent anorexia.

In fed mice, much of the *Salmonella*-induced gastroenteritis is caused by chemokine-mediated recruitment of neutrophils, dendritic cells, inflammatory monocytes and macrophages to the intestinal mucosa where these cells release toxic factors such as reactive oxygen species. Although this inflammatory response promotes bacterial killing, it also leads to widespread tissue damage [52]. Notably, we show that fasting blocks this recruitment of inflammatory cells to the cecum, even when it is heavily infected, as seen in GF mice. This likely occurs by preventing the upregulation of chemokine expression by the intestinal epithelium. Another key pathological response to *Salmonella* invasion is the widespread shedding of both infected and uninfected epithelial cells, following activation of pro-inflammatory pathways including the central immunoregulatory transcription factor NF-κB [53]. We show that NF-κB and its downstream targets (such as *iNos*, *Il-6* and *Tnf-α)* typically induced during *Salmonella* infection, are suppressed during fasting. While we demonstrate that fasting directly affects the inflammatory response of the intestinal epithelium as IEC expression of iNOS is dramatically reduced in fasted infected mice, it cannot be excluded that the reduction of NF-κB expression and its downstream targets could also reflect the limited inflammatory cell recruitment to the mucosa. Furthermore, while higher levels of short chain fatty acids (SCFA) are usually associated with increased tissue protection and anti-inflammatory effects, a few studies have shown that under some circumstances the opposite can be true, and high levels of SCFA can promote inflammation. As such, there is the possibility that the fasting-mediated decrease in SCFA production could have contributed to create an anti-inflammatory environment in the mouse ceca [54,55]. Notably, a reduction in IEC sloughing and preservation of epithelial integrity was also observed in mice that were re-fed following a 48h infection with concurrent fasting, despite visibile inflammatory cell infiltration into the submucosa and the presence of *Salmonella* within the IEC of these re-fed mice. As such, fasting appears to increase disease tolerance, reducing tissue immunopathology independent of pathogen load. Since *Salmonella* infection is known to promote the turnover of IEC, often through inflammasome-mediated pyroptosis, future studies should elucidate whether fasting preferentially inhibits inflammasome activation, or other epithelial cell death pathways [52].

In summary, the data we have obtained indicate that as a food-borne pathogen, *S*. Typhimurium has evolved to benefit from the availability of dietary nutrients within the GI tract to help it compete with resident commensals and infect its host. The protection offered through fasting appears to be mediated by increasing both colonization resistance as well as disease tolerance. Fasting increases colonization resistance through its actions on the microbiome, thereby reducing pathogen fitness by limiting the availability of signals/nutrients required to induce *Salmonella*’s SPI-1 virulence program. Furthermore, fasting increases disease tolerance by suppressing the host inflammatory response through inhibition of NF-κB-mediated inflammatory pathways. As a result, even when *Salmonella* is able to infect its host, the resulting immunopathology is greatly attenuated. These data thus suggest that therapeutic fasting and/or calorie restriction has the potential to beneficially modulate infectious and potentially non-infectious GI diseases.

## Materials and methods

### Ethics statement

All animal experiments were performed in accordance with the Canadian Council on Animal Care (CCAC) guidelines and were under approved ethics protocols by the University of British Columbia’s Animal Care Committee (Protocol Numbers: A15-0211, A15-0206).

### Lead contact

Additional information and requests for resources and reagents should be send to the lead contact for this study: Dr. Bruce A. Vallance (bvallance@cw.bc.ca).

### Materials availability

All unique/stable reagents generated in this study are available from the Lead Contact with a completed Materials Transfer Agreement.

### Mouse strains and diet

WT C57BL/6N mice were obtained from Charles River Laboratories, and were housed for at least one week to acclimatize at BC Children’s Hospital Research Institute (BCCHR) before any procedures were started. Mice were kept under specific pathogen free (SPF) conditions in filter-topped cages, and fed sterilized food and UV-sterilized municipal tap water. For all experiments only female 8-10-week-old mice were used.

Germfree (GF) C57BL/6 mice were obtained by 2-stage embryo transfer and bred at the axenic-gnotobiotic facility at McMaster University were provided in germfree shippers (Taconic) and housed for an initial acclimatization phase of 3 days plus the duration of the experiment (2 days) at BCCHR to minimize contamination. C57BL/6 *Sigirr*^*-/-*^ mice were bred in-house at BCCHR under SPF conditions. Prior to any experiments involving a fasting group, mice were put into new cages with minimal paper-based bedding to reduce coprophagy and avoid access to food pellets hidden in the cage. Even when fasting, mice were never housed singly in order to reduce stress on the animals. Upon transfer to the new cage, mice in the fasting group had no access to food but were given water *ad libitum*. Fasting state was confirmed by blood glucose and ketone measurements. All “fed” mice were given *ad libitum* access to regular chow diet. For microbiome experiments, incoming Charles River mice were housed together in over-sized cages for 5 days before starting any intervention. Mice were then randomly allocated into smaller groups of 3–4 mice per cage for the short duration of the experiment (24h).

### Bacterial strains and culture

For this study a streptomycin-resistant strain of wild-type *Salmonella enterica* serovar Typhimurium SL1344 (WT ST) [56] was used. WT ST carrying the destabilized GFP (GFP[LVA]) reporter plasmids pMPMA3ΔPlac-PprgH-gfp[LVA] [33] (carbenicillin resistant) and WT ST carrying the *luxCDABE* reporter plasmid pCS26-*invF* (kanamycin resistant) were used for assessing virulence gene expression *in vivo*. A *Salmonella* strain expressing the *Photorhabdus luminescens* lux operon on the chromosome (ST *lux*), was used to control for effects of fasting on the luminescence signal, as the luminescence signal in the ST *lux* is constitutively activated—independent of virulence gene expression. WT ST and ST *lux* were cultured in Luria Broth (LB) in a shaker (200 rpm) at 37°C overnight. For ST *lux*-*invF* 50 μg/mL kanamycin and for ST *prgH*-GFP 50 μg/mL carbenicillin were added to the overnight culture respectively.

Additionally, the 81–176 lab strain of wild-type *Campylobacter jejuni* was used in this study [57]. *C*. *jejuni* cultures were grown on Mueller-Hinton agar plates or broth under microaerophilic conditions using anaerojars and CampyGen sachets (Oxoid) at 42°C [36].

### Mouse infections

For oral infections with *S*. Typhimurium C57BL/6 SPF or GF mice were orally gavaged with 100 μl of 200 mg/ml streptomycin (total dose of 20 mg) and 24 h later orally gavaged with 100 μl of a 1:100 dilution of LB overnight culture of *Salmonella* in PBS, which amounts to an infection dose of ~ 2.5 × 10^6^ CFU. Mice were sacrificed between 12 h to 48 h post infection.

For *i*.*v*. infections with *S*. Typhimurium, C57BL/6 SPF mice were injected via the tail vein with 100 μl of 1:10,000 dilution of an LB overnight culture of *Salmonella* in PBS, which amounts to an infection dose of ~ 5 × 10^4^ CFU. Mice were sacrificed between 24 h to 48 h post infection.

For *C*. *jejuni* infections, *Sigirr* ^*-/-*^ mice were orally gavaged with 100 μl of 50 mg/ml vancomycin (total dose of 5 mg) and 5 h later orally gavaged with 100 μl overnight culture of *Campylobacter*, which amounts to an infection dose of ~ 1 × 10^7^ CFU [36]. At the end of each experiment (43 h p.i.), mice were anesthetized by isoflurane and euthanized by subsequent cervical dislocation.

Mouse health status or disease severity were assessed according to the criteria found in Table 1.

**Table 1:** Clinical health score used to assess mouse health status.

Clinical Signs**	Clinical Health Score					
	0	1	2	3*	4	5***
Body Weight	No loss	<5%	5–9%	10–20%	21–24%	>25%
**Appearance/ Activity/ Posture (AAP)**	BAR (Bright/alert/ responsive)	QAR (quiet/alert/ responsive), infrequent hunching	Decreased activity and alertness or hyperactivity, mild piloerection, mild hunching, some limb weakness	Huddled, inactive, circling, mild head tilt, moderate piloerection, tip toe gait, obvious head tilt, obvious weakness	Depressed, hardly moved when handled, shaking or increased aggression, marked piloerection, pale,cool to touch, severe hunching	--
**Hydration**	Normal	--	Some signs of dehydration (Squinting, dull eyes)	Worsening dehydration (sunken dull eyes)	Significant dehydration (sunken, pale eyes, skin tent	--

* A score of 3 in any category or a cumulative score of 6 or higher resulted in increased monitoring, including: weighing the mouse three times a day, supportive care such as SQ fluids (0.5cc, no more than every 12h).

** A cumulative score of >8 requires either immediate refeeding or euthanasia.

***Body weight loss over 25% requires either immediate refeeding or euthanasia.

### Blood glucose and ketone measurement

At the end of each experiment, before euthanizing mice, one drop of blood was collected from the tail vein of mice and directly applied to a GE200 glucose measurement test strip that was read by the GE200 glucose monitor (GE, Auto Control Medical). Blood glucose was measured before administering anesthesia to prevent spikes in blood glucose due to stress and isoflurane treatment [58]. For measuring blood concentration of the ketone body β-hydroxybutyrate (βHB), 0.5–1 ml of blood were collected with cardiac puncture after deeply anesthetizing mice with isoflurane followed by immediate cervical dislocation. Blood was added to tubes containing K2-EDTA (BD Microtainer 365974), centrifuged at 1000 rcf for 12 min at 4°C. Serum was then transferred into an Eppendorf tube and kept at -20°C until further use. βHB concentration in serum was measured using the Beta-Hydroxybutyrate LiquiColor Assay (StanBio) according to the manufacturer’s instructions, scaled down to 5 μl sample per well. Colorimetric change was measured at 505 nm and background absorbance was subtracted from final reading to account for potential hemolysis.

### Tissue collection

After euthanasia, the abdominal cavity of mice was opened, organs of interest removed and small pieces of tissues (~ 0.5 cm x 1cm) were cut from the respective organs (cecum, colon, liver, spleen). For histology preparation, tissues were immediately placed in 10% neutral buffered formalin (Fisher) for 24 h at 4°C. Tissues were then transferred to 70% ethanol and stored for 1–5 d until they were paraffin embedded. Paraffin blocks were cut into 5 μm sections and mounted on microscope glass slides. For RNA analysis, stool was removed from intestinal tissues and the tissue pieces were immediately placed into RNAlater (Qiagen, Gaithersburg, MD), kept at 4°C overnight and then stored at -80°C until further processed.

For protein analysis via immunoblotting, cut tissues were washed in ice-cold PBS and then immediately placed into tubes with 1 ml ice-cold RIPA buffer containing cOmplete protease inhibitor (Roche) and PhosSTOP phosphatase inhibitor (Roche). Tissues were homogenized by bead beating in a Mixer Mill MM 400 (Retsch, Haan, Germany) for 6 min at 30 Hz. Then, samples were centrifuged for 20 min at 13000 rpm, at 4°C. The supernatant was collected and stored at -80°C until further analysis. For protein analysis via ELISA, cut tissues were thoroughly washed in ice-cold PBS and then placed into 1 ml PBS containing cOmplete protease inhibitor (Roche). Tissues were homogenized as described above and then centrifuged for 10 min at 12000 rpm, at 4°C. The supernatant was collected and stored at -80°C until further analysis.

### Bacterial counts

After samples were collected for histology, RNA and protein analysis as described above, the remaining organ tissue was washed extensively in PBS and then placed into a previously weighed 2 ml Eppendorf tube containing a metal bead (Qiagen) and weighed again. 1 ml PBS was added to each tube and tissues were homogenized by bead beating in a Mixer Mill MM 400 (Retsch) for 6 min at 30 Hz at room temperature. Homogenized tissues were then 1:10 serially diluted in PBS and in triplicates plated onto agar plates. For WT ST LB agar plates containing 100 μg/ml streptomycin (plus 50 μg/mL carbenicillin for ST *lux-invF* and 50 μg/mL kanamycin for ST *prgH-*GFP respectively) were used. Plates were incubated overnight at 37°C and CFUs counted the next day. For *C*. *jejuni* enumeration, serially diluted and homogenized tissues were plated onto *Campylobacter*-specific agar plates containing Karmali selective supplements (Oxoid) and incubated under microaerobic conditions for 24–48 h at 37°C [59]. Bacterial counts were normalized to tissue weight.

### pH measurement

To measure stomach pH, each mouse stomach was removed, and surgically dissected into upper and lower gastric contents as well as stomach tissue and collected into 1.5mL microcentrifuge tubes and kept on ice. A sterile inoculating loop was used to collect gastric contents. The loop with either gastric contents or the mucosal tissue was directly dabbed onto pH paper (pH Test Strips 0.0–6.0, VWR Chemicals BDH). Using the pH scale provided by the manufacturer, pH was assessed when the paper was still wet from the sample to ensure the most accurate measurement.

### Histology and pathology scoring

Paraffin-embedded cross sections of organs (5 μm) were stained with haematoxylin and eosin (H&E) as previously described [59]. Tissue pathology was assessed by light microscopy using a Zeiss AxioImager microscope and scored by three observers blinded to the experimental groups. The scoring matrix (with a max. score of 21) was adapted from Barthel *et al*. [16] and includes

- *submucosal edema* (0  =  no change; 1  =  mild; 2  =  moderate; 3  =  severe)
- *crypt hyperplasia* (% change compared to height of control with 0  =  no change; 1  =  1–50%; 2  =  51–100%; 3 >100%)
- *epithelial integrity* (0 = no pathological changes detectable; 1 = few cells sloughed, epithelial surface rippled; 2 = moderate amount of cells sloughed, epithelial surface is damaged in some areas; 3 = large amounts of cell sloughing, epithelial surface severely damaged; 4 = epithelium completely destroyed, no crypt structure recognizable, ulceration)
- *submucosal polymorphonuclear leukocytes (PMN)* and
- *mucosal PMN* infiltration per 400× magnification field (0 <5 cells; 1 = 5–20 cells; 2 = 21–50 cells; 3 = 51–100 cells, 4>100 cells/field)

H&E stained tissues were viewed and images captured on a Zeiss AxioImager microscope connected to an AxioCam HRm camera running the AxioVision software (Version 4.4).

### Immunofluorescence staining

Paraffin-embedded tissue sections (5 μm) were deparaffinized by heating the slides for 20 min in a water bath, followed by xylene immersion, and then rehydration through an ethanol to water gradient. Then, slides were placed in antigen retrieval buffer (0.1 M citric acid monohydrate with 0.05% Tween 20, pH = 6) and heated in a steamer for 30 min. After a 30 min cool down phase at RT, slides were washed in distilled water. Tissues then underwent a 15 min permeabilization step at RT with permeabilization buffer (PBS, 0.1% Triton X-100 and 0.05% Tween 20) in order to enhance intracellular antibody binding.

Following that, slides were blocked in PBS containing 5% serum from either donkey or goat, 0.1% Triton X-100 and 0.05% Tween 20. Slides were then incubated with the primary antibodies diluted in blocking buffer at 4°C overnight. For detection of biotinylated antibodies the Endogenous Biotin-Blocking Kit (E-21390, Invitrogen) was used according to the manufacturer’s recommendations prior to the regular blocking step.

After primary antibody incubation, slides were washed three times with PBS and then incubated with the appropriate secondary antibody diluted in blocking buffer for 1 h at RT. The primary and secondary antibodies that were used can be found in Table 2.

**Table 2:** Antibodies used in this study.

ANTIBODIES	SOURCE	IDENTIFIER
*Salmonella* O antisera Group B Factors 1, 4, 12, 27	Difco BD	Cat#229731
mouse anti-F4/80	Bio-Rad	MCA497GA
mouse anti-Ly-6G	BD	Cat#551459
mouse anti-Cytokeratin-19	Santa Cruz Biotechnology	sc-33111
mouse anti-iNOS	Millipore	06–573
biotinylated goat anti-GFP	GeneTex	GTX26658
mouse-anti-LAMP-1	DSHB	1D4B
biotinylated anti-*Campylobacter jejuni*	Abcam	ab53909
mouse-anti-Mucin 2	Santa Cruz Biotechnology	sc-15334
AlexaFluor 568-conjugated goat anti-rat IgG	ThermoFisher/Invitrogen	A-11077
AlexaFluor 568-conjugated donkey anti-rabbit IgG	ThermoFisher/Invitrogen	A10042
AlexaFluor 568-conjugated streptavidin	ThermoFisher/Invitrogen	S11226
AlexaFluor 488-conjugated goat anti-rat IgG	ThermoFisher/Invitrogen	A-11006
AlexaFluor 488-conjugated donkey anti-goat IgG	ThermoFisher/Invitrogen	A-11055
AlexaFluor 488-conjugated donkey anti-rabbit IgG	ThermoFisher/Invitrogen	A-21206
mouse anti-Phospho-NF-κB	Cell Signaling Technology	Cat#3033
mouse anti-total NF-κB p65 (D14E12) XP	Cell Signaling Technology	Cat#8242
mouse anti-β-actin	Santa Cruz Biotechnology	sc-1616-R
anti-rabbit IgG	Cell Signaling Technology	Cat#7074

Stained tissues were washed twice with PBS and once with distilled water and then mounted using ProLong Gold Antifade reagent that contains 4′, 6′-diamidino-2-phenylindole (DAPI) to stain DNA. Stained tissues were viewed and images captured on a Zeiss AxioImager microscope connected to an AxioCam HRm camera running the AxioVision software (Version 4.4).

### Intracellular salmonella scoring

Cecal tissue sections previously immunostained for *Salmonella*, Cytokeratin 19 and DAPI, as described above, were used for scoring. Two observers blinded to the experimental group assessed the presence of *Salmonella* in IEC in cecal tissue as well as the extent of epithelial damage and apical IEC shedding at a magnification of x400 using a Zeiss AxioImager microscope. The scoring matrix (with a max. score of 8) was partially adapted from Barthel *et al*. [16] for epithelial damage assessment and scored as follows:

- *epithelial integrity* (0 = tissues which look pristine—on average less than 1 sloughed cell per 400X high power field; 1 = tissue has not gone through overt crypt hyperplasia, 1–5 cells sloughed per high power field; 2 = tissue is overtly hyperplastic and/or more than 5 sloughed/sloughing cells per high power field; 3 = loss of crypt structure, more than 10 sloughed cells per high power field)
- *Intracellular Salmonella* (0 = no *Salmonella* in tissue, a few salmonella in lumen; 1 = occasional *Salmonella* in epithelial cells—ie. scanning an entire cross section, at least 1 intracellular *Salmonella*—a few *Salmonella* in lumen; 2 = 1–5 *Salmonella* in epithelial cells in every cross section—a few *Salmonella* in lumen; 3 = 5–10 *Salmonella* inside IEC in every cross section and a few *Salmonella* in lumen; 4 = more than 10 *Salmonella* inside IEC in some fields and a large number of *Salmonella* in sloughed IEC in lumen (essentially a patchy form of score 5); 5 = every high power field has Salmonella inside epithelial cells (more than 10), adherent to epithelial cells or signs of epithelial cells bursting because of infection along with large numbers of *Salmonella* and sloughed IEC in lumen)

### RNA extraction and quantitative real-time PCR for gene expression analysis

For RNA preservation, tissues were collected and stored as described above under “tissue collection”. Total RNA was extracted using the RNeasy Plus Mini kit (Qiagen). To homogenize tissues, each piece of tissue was placed in a 2 ml Eppendorf tube containing a metal bead. 600 μl of RLT buffer containing β-mercaptoethanol were added. Tissues were then homogenized by bead beating in a Mixer Mill MM 400 (Retsch) for 6 min at 30 Hz. Then, samples were centrifuged for 3 min at 13000 rpm. The supernatant was transferred onto gDNA elimination spin column. Protocol was further followed according to the manufacturer’s instructions. RNA yield was quantified using a NanoDrop spectrophotometer (ND1000). 500 ng of RNA was reverse-transcribed to complementary DNA (cDNA) using 5X All-In-One RT MasterMix (abm -Applied Biological Materials, Richmond, Canada) according to the manufacturer’s instructions. For quantitative real-time PCR (qPCR), cDNA was diluted 1∶5 in RNase/DNase free water. 5 μl of the diluted cDNA was then added to PCR reaction master mix containing 10 μl SsoFast EvaGreen Supermix (Biorad) as well as the appropriate primer pair diluted in RNase/DNase free water, bringing the total reaction mix to 20 μl with a final primer concentration of 0.3 μM. qPCR was carried out using the CFX Connect Real-time PCR Detection System (Biorad). Primer specificity was confirmed by melting point analysis, primers were optimized to perform with efficiencies between 90 and 110% as confirmed by running separate standard curves for each primer and organ. Gene expression was analyzed with the CFX Maestro software (Bio-Rad). All genes were normalized to three housekeeping genes at once (*Rplp0*, *Tbp*, *eEF-2*). Expression was further normalized by setting the average expression of uninfected fed WT mice to 1. The resulting ratios were log10 transformed for statistical analysis and thus presented as relative expression values (log-fold over control). Primer sequences can be found in the Table 3.

**Table 3:** Primers used for qPCR.

Gene	Primer sequence
*Il-1β*	F: CAG GAT GAG GAC ATG AGC ACC R: CTC TGC AGA CTC AAA CTC CAC
*Il-6*	F: GAG GAT ACC ACT CCC AAC AGA CC R: AAG TGC ATC ATC GTT GTT CAT
*Tnf-α*	F: CAT CTT CTC AAA ATT CGA GTG ACA A R: TGG GAG TAG ACA AGG TAC AAC CC
*iNos*	F: GGTGAAGGGACTGAGCTGTT R: ACGTTCTCCGTTCTCTTGCAG
*Ifn-γ*	F: ATG AAC GCT ACA CAC TGC ATC R: CCA TCC TTT TGC CAG TTC CTC
*Mcp-1*	F: TGATCCCAATGAGTAGGCTGGAG R: ATGTCTGGACCCATTCCTTCTTG
*Cxcl1*	F: ATC CAG AGC TTG AAG GTG TTG R: GTC TGT CTT CTT TCT CCG TTA CTT
*Cxcl2*	F: CCT GCC AAG GGT TGA CTT CA R: TTC TGT CTG GGC GCA GTG
*RegIII-β*	F: ACAAGATGCTGCCTCCAACAG R: AGGGAGTCTTCACCTTGAACC
*RegIII-γ*	F: ATGCCCCATCTTCACGTAGC R: TGGCAGGCCATATCTGCATC
*Rplp0*	F: TCATCCAGCAGGTGTTTGACA R: GGCACCGAGGCAACAGTT
*Tbp*	F: ACC GTG AAT CTT GGC TGT AAA R: GCA GCA AAT CGC TTG GGA TTA
*eEF-2*	F: TGT CAG TCA TCG CCC ATG TG R: CAT CCT TGC GAG TGT CAG TGA

### IL-1β quantification by Enzyme-linked Immunosorbent assay (ELISA)

Tissues were collected and homogenized as described above. IL-1β was measured using the mouse IL-1β ELISA set (BD Biosciences, San Diego, USA) according to the manufacturer’s protocol using MaxiSorp 96-well plates (Nunc, Thermo Fisher Scientific) and 100 μl of sample.

### Western blotting

Protein lysates were extracted as described above under “tissue collection”. Protein concentration was measured with the Pierce 660nm Protein Assay Kit (Thermo Fisher Scientific). For each tissue, 30 μg of protein was prepared for SDS PAGE by adding 4x Laemmli Sample Buffer (Biorad) containing appropriate amount of 2-mercaptoethanol. Samples were then heated at 80°C for 10 min for denaturation. Following that, samples were loaded onto 4–15% Mini-PROTEAN TGX Stain-Free Protein Gels (Biorad). Proteins were separated in Tris/Glycine/SDS electrophoresis buffer (Biorad) in a Mini-PROTEAN Tetra Vertical Electrophoresis Cell (Biorad), run at RT for 20 min at 200 V constant. Proteins were then transferred onto Immun-Blot Low Fluorescence PVDF Membrane (Biorad) in a Trans-Blot Turbo Transfer System (Biorad) using a Ready-to-assemble transfer kit (Biorad) for 3 min at 2.5 A constant. Immediately after transfer, membranes were imaged with a ChemiDoc Imaging System (Biorad) using the Stain-Free setting for total protein quantification and subsequent normalization. Membranes were then blocked in 5% BSA in TBS-T for 1 h at RT and incubated overnight at 4°C with the primary antibody against Phospho-NF-κB (#3033, Cell Signaling Technology, 1:1000), total NF-κB p65 (D14E12) XP (#8242, Cell Signaling Technology, 1:1000) and anti-β-actin (sc-1616-R, Santa Cruz Biotechnology, 1:2000). Following a washing step in TBS-T, membranes were then incubated with the HRP-linked secondary antibody anti-rabbit IgG (7074; 1:2000; Cell Signaling Technology) for 1 h at RT. Restore Western Blot Stripping Buffer (ThermoFisher Scientific) was used according to manufacturer instructions to allow for detection of Phospho-NF-κB and total NF-κB on the same membrane. Chemiluminescence signal was measured with the ChemiDoc Imaging System (Biorad) using ECL Plus Western Blotting Substrate (Pierce, ThermoFisher Scientific).

### Microbiome analysis

#### Cecal content collection and DNA extraction

Content was collected from mouse ceca, transferred into Eppendorf tubes and immediately frozen at -80°C. Samples were then shipped on dry ice to the Children’s Hospital of Philadelphia (CHOP) Microbiome Center were DNA was extracted and samples were sequenced. DNA was extracted using the DNeasy PowerSoil Kit (Qiagen) following the manufacturer’s instruction including the optional heating step at 70°C for 10 min before bead beating.

#### Library prep and sequencing

Libraries were generated using a two-step PCR. First, the 16S V3V4 region was amplified in 25 μl quadruplicate reactions with the Q5 Hot Start High-Fidelity DNA polymerase (New England BioLabs) using a primer pair selected from Klindworth et al. [60] that also contained overhanging Illumina adapter sequences (protocol outline and primer sequences can be found here:

https://support.illumina.com/documents/documentation/chemistry_documentation/16s/16s-metagenomic-library-prep-guide-15044223-b.pdf.) Quadruplicate reactions were then pooled and DNA cleaned up using solid-phase reversible immobilization (SPRI) on carboxylated paramagnetic beads (GE Healthcare, 65152105050250) at a 1:1 ratio. Secondly, Nextera XT barcodes (Illumina) were added in another PCR step. Libraries were cleaned up again using SPRI beads and subsequently quantified with PicoGreen (P7589, Invitrogen, ThermoFisher Scientific). Equimolar quantities of each sample were pooled and sequenced on the Illumina MiSeq using 2x300 bp chemistry (MiSeq Reagent Kit v3 MS-102-3003, Illumina).

#### Microbiome computational analysis

Illumina sequencing data from each experiment were processed and analyzed using QIIME2 v2019.7 [61]. Paired reads were trimmed to remove low-quality bases (Q < 20), adapter, and primer sequences using the Cutadapt module within QIIME2 [62]. Resultant reads were denoised and merged using DADA2 [63]. A phylogenetic tree was constructed by QIIME2 (phylogeny align-to-tree-mafft-fasttree command) with midpoint rooting. The reads were assigned to species-equivalent amplicon sequence variants (ASVs) at 99% similarity by using the feature-classifier classify-sklearn algorithm against the Silva_132 release reference sequences [64]. Samples with fewer than 1,000 ASV-assigned sequences were omitted. The filtered ASV table, rooted tree, and taxonomy QIIME2 objects (along with the metadata table) were imported into R as a phyloseq object using qiime2R v0.99. Phyloseq v1.32.0 [65] was used for alpha- and beta-diversity calculations and principal coordinates analysis plots (ellipses represent 95% confidence intervals). Differential abundance was assessed using beta-binomial regression within the corncob package (v0.2.0) [66].

### Droplet digital PCR for quantification of total bacteria and bacterial virulence gene transcription

In order to quantify the total amount of the bacterial DNA present in DNA isolated from equal amounts of cecal content of mice, droplet digital PCR (ddPCR) was performed using UniF340 forward and UniR514 reverse primer. DNA was extracted as described above. For RNA extraction from cecal contents, nucleic acids were isolated using the MagMAX Microbiome Ultra Nucleic Acid Isolation Kit on a KingFisher Duo Prime system. Nucleic acids were digested with InvitrogenTURBO DNA-*free* Kit following the manufacturer’s instruction for “rigorous DNAse treatment”. RNA was then reverse-transcribed into cDNA with OneScript Hot cDNA Synthesis Kit (Abcam) using random primers.

For both genomic DNA and cDNA quantification a 25 μL ddPCR reaction was prepared with 2 μL of sample DNA, 10 μL of EvaGreen Supermix (BioRad, Hercules, CA, United States), 0.50 μL of each primer at 10 μM, and 12 μL of PCR grade water. Samples were placed into a QX200 Automated Droplet Generator (BioRad, Hercules, CA, United States) which produces uniform droplets for each sample during the partitioning process. The PCR step was performed in a Bio-Rad C1000 thermal cycler using the following protocol: 1 cycle at 95°C for 5 minutes, 40 cycles at 95°C for 130 seconds and 63°C (gDNA) or 60°C (cDNA) for 1 minute, 1 cycle at 4°C for 5 minutes, and 1 cycle at 90°C for 5 minutes all at a ramp rate of 2.5°C/second. Following PCR amplification, the PCR plate was placed in a QX200 Droplet Reader (BioRad, Hercules, CA, United States), which analyzed each droplet individually using the FAM color detection system. Positive and negative droplets were spaced out based on their individual fluorescence and the data analysis was conducted using the Bio-Rad Quantasoft software. Primer sequences can be found in the Table 4.

**Table 4:** Primers used for droplet digital PCR.

Gene	Primer sequence
*UniF340 UniR514*	F: ACTCCTACGGGAGGCAGCAGT R: ATTACCGCGGCTGCTGGC
*rrsA (16S)*	F: TCTGGGAAACTGCCTGATGGAGG R: CGTAGGAGTCTGGACCGTGTCTC
*invF*	F: GGTCTCCTGATACTGGTGCG R: AGCCCGGAAGCATGGTTTAT
*prgH*	F: GCCAGCTGCGGATAATAGGT R: TGTCGCTGCGCAAAATGAAA
*sipB*	F: TGAGACAGCGAAACATCGCC R: GGAACAAAGTCCGGCGAGAG
*sipC*	F: TGCCGTCGTTTTAGCTGCAT R: AAACCCAGTTACGCGAGCAG
*sicA*	F: TTCAGTTGGCATACTGCCGC R: GAAGGCGCCACGCTAAAAGA

### Short chain fatty acid (SCFA) quantification

Acetic, propionic and butyric acid were extracted from cecal content using direct-injection gas chromatography (GC) as described previously [67]. Briefly, ~30 mg of cecal content was homogenized with isopryl alcohol and 2-ethylbutyric acid (0.01% v/v) as an internal standard. Homogenates were centrifuged and the clear supernatant was injected into a Trace 1300 Gas Chromatograph, equipped with a flame-ionization detector and an AI1310 autosampler (Thermo Scientific, Walkham, MA, USA), in splitless mode. Data were analyzed with the Chromeleon 7 software (Bannockburn, IL, USA). Fine separation of SCFA was confirmed by the complete separation of the volatile-free acid mix (Sigma, Oakville, ON, Canada). Data are presented as μmol of SCFA per g of dry weight cecal content.

### Generation of *Salmonella* strains expressing the *Photorhabdus luminescens* lux operon

To generate the *invF-luxCDABE* transcriptional fusion, a 423 bp fragment spanning the regulatory region of *invF*, the first gene of the inv/spa operon, from position -312 to + 111 with respect to its transcriptional start site, was amplified by PCR using oligonucleotides *invF-lux*-Fw (CTATCTCGAGCAGAAGAATGAGGCGCCATG) and *invF-lux*-Rv (CATTGGATCCCACGTCTGTATAAACCATGC) and *S*. Typhimurium SL1344 chromosomal DNA as the template. The PCR product was purified with the High Pure PCR product purification kit (Roche) and digested with XhoI and BamHI and then ligated with T4 DNA ligase (Fermentas, Pittsburgh, PA) to plasmid pCS26-*Pac* [68], a plasmid carrying the promoterless *luxCDABE* operon of *Photorhabdus luminescens*, digested with the same enzymes. The ligation reaction was transformed into *E*. *coli* DH5α and the derived construct pCS26-*invF* was confirmed by DNA sequencing. *E*. *coli* DH5α containing the pCS26-*invF* plasmid was grown overnight at 37°C in LB medium containing 50 μg/ml kanamycin. The pCS26-*invF* plasmid was isolated with the QIAprep Spin Miniprep Kit (Qiagen). The plasmid was then transformed into electrocompetent wild-type *Salmonella enterica* serovar Typhimurium SL1344 closely following the protocol described by Bayoumi and Griffiths [69].

To generate the *Salmonella* strain expressing the *Photorhabdus luminescens* lux operon on the chromosome (ST *lux*), a triple mating involving wildtype *S*. Typhimurium SL1344, *E*. *coli* MFDλpir containing plasmid pMAC5-lux, and *E*. *coli* MFDλpir containing a helper plasmid pTNS2 [70] was performed on LB agar containing diaminopimelic acid (DAP). In the plasmid pMAC5-lux, the lux operon was expressed under the control of the promoter PLtetO [71,72] and cloned into pMAC5, a chloramphenicol-marked Tn7 delivery vector [73]. Conjugants were selected by plating 24 h conjugation mixture onto LB agar containing chloramphenicol but lacking DAP, and screened for the proper Tn7 transposition as before [70]. All bacterial strains were grown from single colonies on LB plates, and cultured in 5 ml of LB broth without antibiotics, or with chloramphenicol (30 μg/ml), Ampicillin (100 μg/ml), or DAP (0.3 mM) at 37°C overnight with shaking at 200 rpm.

### Imaging luminescence in *Salmonella*-infected tissues *ex vivo*

Fed and fasted mice infected with WT ST carrying the luciferase reporter plasmid pCS26-*invF* were euthanized simultaneously at 15 h post infection, abdominal cavity was opened and cecum and colon (in one piece) was immediately taken out and placed on black cardboard paper. Bioluminescence signal emanating from tissues was imaged simultaneously for fed and fasted mice with an *in vivo* imaging system (Ami-X; Spectral Instruments Imaging, AZ), Pictures were taken and analyzed with AURA Imaging Software (Spectral Instruments Imaging).

### Quantifications and statistical analysis

Statistical analyses were performed in Microsoft Excel (Mac 2019), GraphPad Prism 9 and R v3.6.1. All data sets were checked for normal distribution with Shapiro-Wilk test. Non-normally distributed data was either log-transformed, where log-normal distribution was indicated, or non-parametric tests were used otherwise. For all CFU counts a pseudocount of 0.5 was added to allow for log-transformation of zero counts. For data with normal or lognormal distribution, an unpaired two-tailed Student’s t test, a multiple t-test with Holm-Sidak correction for multiple testing or one-way or two-way analysis of variance (ANOVA) followed by Tukey’s multiple comparisons post test, were used to determine the statistical significances. Results are presented as the mean value ± standard deviation (SD) unless otherwise indicated. Box plots show the median and 25th and 75th percentile with whiskers depicting largest and smallest value of the data set. For histopathological analysis, agreement across scorers was calculated using Kendall’s coefficient of concordance W. Significance levels used are * p<0.05, ** p<0.01, *** p<0.001, **** p<0.0.0001. All immunofluorescence images were edited and look up tables were assigned for better visibility using Fiji distribution of ImageJ v2.0.0-rc-69/1.52i. Figures were designed and assembled using Adobe Illustrator v24.1.3. Microbiome data was analysed as described above under “Microbiome Computational Analysis”.

## Supporting information

S1 Fig Establishing a safe fasting regimen in mice and excluding mechanisms not responsible for the effects of fasting on pathogen load. (A) Body weight loss over time during fasting and re-feeding in un-infected control C57BL/6 mice. Critical weight (25% loss of inital body weight) refers to animal ethics protocol limit that cannot be exceeded during any experiment. (n = 7) (B) Glucose levels in mouse whole blood in control un-infected ad libitum fed or 48h fasted mice. (C) β-hydroxybutyrate levels in mouse serum in control un-infected ad libitum fed or 48h fasted mice. (D) Disease/Health Score assessing impact of 24h infection ± fasting on mice. AAP = Activity, Appearance, Posture. Detailed information on scoring matrix can be found in the Materials and Methods section. (E) *S*. Typhimurium CFU per g liver or spleen tissue 24h p.i. ± 48h of fasting. (see Fig 1A for experimental timeline). (F) qPCR analysis of two antimicrobial-peptide genes in *S*. Typhimurium-infected mouse ceca expressed as fold change over fed ctrl. Fed ctrl refers to uninfected ad libitum fed C57BL/6 SPF mice. (G) pH of stomach content and tissue of uninfected mice 24h after oral strep treatment ± 24h of fasting. (H) *S*. Typhimurium CFU per g tissue 12h p.i. ± 36h of fasting. (I) Invasion score quantifying *S*. Typhimurium presence in IEC by analyzing immunofluorescently stained cecal sections at 24h p.i. Data identical to Fig 1I–but fasting group was split up into mice with high (≥ CFU 10^6^) or low (CFU 10^3^–10^5^) pathogen burdens. **** p < 0.0001, ** p < 0.01, Significance levels calculated by unpaired Student’s t test (B,C), multiple t-test (G), Mann-Whiteney-Wilcoxon test (D, E, H, I)–each with Holm-Sidak correction, two-way ANOVA with Tukey post test (F). Error bars shown as ± SD for (A-C, F-G), and as box plot with min-max whiskers (D, E, H, I).
(TIF)

S2 Fig Re-feeding after fasting differentially affects the pathogen and the host. (A) Experimental timeline of infection and diet regimen. Streptomycin-pretreated SPF mice were orally gavaged with ~ 2.5 × 10^6^ colony-forming units (CFU) *S*. Typhimurium and sacrificed 48h p.i. Mice were either fed throughout the whole experiment (blue), or fasted for the first 48h and re-fed the following 24h of the experiment (green). (C, D) Representative H&E-stained cecal sections of mice at 48h p.i. with *S*. Typhimurium (see S2A Fig for experimental timeline). Scale bar 200 μm (DC), 50 μm (D). (B) Histopathological analysis of cecal tissue H&E sections (as shown in C-D, see Materials and Methods for scoring criteria). Agreement among raters ensured by Kendall’s coefficient of concordance WT = 0.8385 (n ≥ 7). (E) Representative immunofluorescence staining of *S*. Typhimurium and IEC on paraffin embedded cecal sections at 48h p.i. ± 48 h of fasting. Sections were stained using DAPI to detect DNA (blue), anti-*Salmonella*-LPS (SLA, red) to visualize *S*. Typhimurium and anti-cytokeratin 19 (CYT19, green) to stain IEC. Scale bar 50 μm. (F) Enumeration of *S*. Typhimurium CFU per g cecal tissue and stool (combined) of mice at 48h p.i. (G) Invasion score quantifying *S*. Typhimurium presence in IEC by analyzing immunofluorescently stained paraffin-embedded cecal sections at 48h p.i. ± 48h of fasting shown in (E) (see Materials and Methods for scoring criteria). Agreement among raters ensured by Kendall’s coefficient of concordance WT = 0.939 (n ≥ 5). For (B), (F) and (G) data from multiple independent experiments were pooled. **** p < 0.0001, *** p < 0.001, ** p < 0.01, * p < 0.05, ns = not significant. Significance levels calculated by unpaired Student’s t test (B), Mann-Whitney-Wilcoxon test with Holm-Sidak correction (F, G). Error bars shown as ± SD (B); box plot with min-max whiskers (F, G).
(TIF)

S3 Fig **Fasting does not affect the efficacy of streptomycin but changes cecal microbiome composition** (A) Experimental timeline of streptomycin treatment and fasting/infection regimen. SPF C57BL/6 mice were orally gavaged with 20 mg of streptomycin and fasted for the next 24h. A second group was given streptomycin, fed for another 5h and fasted for the following 19h. Mice were then orally infected with ~ 2.5 × 10^6^ CFU *S*. Typhimurium and sacrificed at 24h p.i. (B) Enumeration of *S*. Typhimurium CFU per g cecal tissue and stool (combined) of mice at 24h p.i. (see S3A Fig for experimental timeline, data for fasted mice is the same as shown in Fig 1J). Data pooled from multiple independent experiments and shown as box plot with min-max whiskers. Significance levels determined by multiple Mann-Whitney-Wilcoxon test with Holm-Sidak correction. (C,D) Representative H&E stained cecal sections of fed.fasted mice (yellow group) at 24h p.i. (see S3A Fig for experimental timeline). Scale bar 200 μm (C), 50 μm (D). (E) Relative abundance of bacteria from the cecal content of SPF mice (for experimental timeline see Fig 3A) on phylum and genus levels. Taxa that were less than 1% abundant were grouped together for better visibility.(F-H) Relative abundance of Clostridia (F), *Enterobacteriaceae* (G) and *Akkermansia* (H) across groups.
(TIF)

S4 Fig **Fasting protects germfree mice from gastroenteritis but not from epithelial cell invasion** (A,B) Representative H&E-stained cecal sections of GF mice 24h post-streptomycin treatment, uninfected ± 24h of fasting. Scale bar 200 μm (C), 50 μm (D). (C) *S*. Typhimurium CFU per g liver or spleen tissue 24h p.i. ± 48h of fasting in GF mice. (see Fig 4A for experimental timeline). (D) qPCR analysis of two antimicrobial-peptides in *S*. Typhimurium-infected mouse ceca expressed as fold change over fed ctrl. Fed ctrl refers to uninfected ad libitum fed C57BL/6 GF mice. For (C) and (D) data from multiple independent experiments were pooled. * p < 0.05, ns = not significant. Significance levels calculated by multiple Mann-Whitney-Wilcoxon test with Holm-Sidak correction (C), or two-way ANOVA with Tukey post test (D). Error bars shown as ± SD (C), box plot with min-max whiskers (D).
(TIF)

S5 Fig Fasting suppresses mucosal NF-κB expression. (A) Representative immunoblot detecting phospho and total NF-κB p65/RelA protein in whole cecal tissue lysates of SPF and GF mice at 24h p.i. with *S*. Typhimurium ± 48h of fasting. Lanes represent data from individual mice. Shown here is the whole PVDF membrane (cut into respective sizes to probe for target), cropped bands of interest are shown in Fig 5F. (B) Total protein stain of whole PVDF membrane (used for Figs 5F and S5A) additionally confirming comparabale protein loading in all lanes.
(TIF)

S6 Fig *S*. Typhimurium is unable to effectively induce SPI-1 in the intestines of fasted SPF mice but retains this ability in fasted GF mice. (A-E) Streptomycin-pretreated mice were orally infected with the respective *S*. Typhimurium strain, at a dose of ~ 2.5 × 10^8^ CFU. Mice were euthanized and intestines imaged at 15h p.i. ± 39h of fasting. Bioluminescence signals were measured in logarithmic units of light (photons/s/cm^2^/sr), divided by log_10_CFU of *Salmonella* pathogen burden. Resulting ratios are expressed as log_10_ of percent change fed over fasted. (A) Representative macroscopic *ex vivo* images and corresponding quantification of bioluminescence signal in SPF fed and fasted mouse intestines infected with a *Salmonella* strain expressing the *Photorhabdus luminescens* lux operon on the chromosome (ST *lux*). Images show magnitude of bioluminescence signal corresponding to presence of total *Salmonlla* numbers. (B,C) Representative macroscopic *ex vivo* images and corresponding quantification of SPF (B) and GF (C) mouse intestine showing magnitude of bioluminescence signal corresponding to expression of the *Salmonella* SPI-1 *invF-luxCDABE* transcriptional fusion. Images in (C) are the same data as shown in Fig 6B. (D,E) Representative images and corresponding quantification of SPF mouse washed cecum (D) and stool (E) bioluminescence signal imaged within Eppendorf tubes. Bioluminescence signal corresponding to expression of the *Salmonella* SPI-1 *invF-luxCDABE* transcriptional fusion. For (A, B, D, E) data from multiple independent experiments were pooled. **** p < 0.0001, *** p < 0.001, ns = not significant. Significance levels calculated by unpaired Student’s t test. Error bars shown as ± SD.
(TIF)

S7 Fig Fasting prevents *Campylobacter jejuni* expansion in the ceca of *Sigirr* ^*-/-*^ mice. (A) Experimental timeline of infection and fasting regimen and concurrent body weight loss. SPF *Sigirr*^*-/-*^ mice were orally gavaged with 5 mg vancomycin and 5h later orally infected with ~ 1 × 10^7^ CFU *C*. jejuni and sacrificed 43h p.i. Mice were either fed (pink) or fasted (brown) throughout the whole experiment. (B) β-hydroxybutyrate levels in mouse serum in fed or 48 h fasted *Sigirr*^*-/-*^ mice 43h p.i. with *C*. jejuni. (C, D) Representative H&E-stained cecal sections of mice at 43h p.i. with *C*. jejuni (see S7A Fig for experimental timeline). Scale bar 200 μm (C), 50 μm (D). (E, F) Representative immunofluorescence staining of *C*. jejuni on paraffin embedded cecal sections at 24h p.i. ± 48h of fasting. Sections were stained using DAPI to detect DNA (blue), anti-Mucin-2 to stain goblet cells filled with mucus, anti-*C*. jejuni (red) and anti-LAMP-1 (LAMP1, green) to stain for bacteria in *Campylobacter*-containing vacuoles (indicated by white arrows). Scale bar 50 μm (E), 10 μm (F). (G) Enumeration of *C*. jejuni CFU per g tissue or stool at 43h p.i. For (A) and (G) data from multiple independent experiments were pooled. **** p < 0.0001, ** p < 0.01. Significance levels calculated by unpaired Student’s t test (B), multiple t-test (A) or Mann-Whitney-Wilcoxon test (G) with Holm-Sidak correction. Error bars shown as ± SD (A,B), box plot with min-max whiskers (G).
(TIF)
