# Supplementary figures and images for: Fasting increases microbiome-based colonization resistance and reduces host inflammatory responses during an enteric bacterial infection
Source: PLoS Pathog. 2021 Aug 5;17(8):e1009719. doi: 10.1371/journal.ppat.1009719 (PMC8341583; doi:10.1371/journal.ppat.1009719)

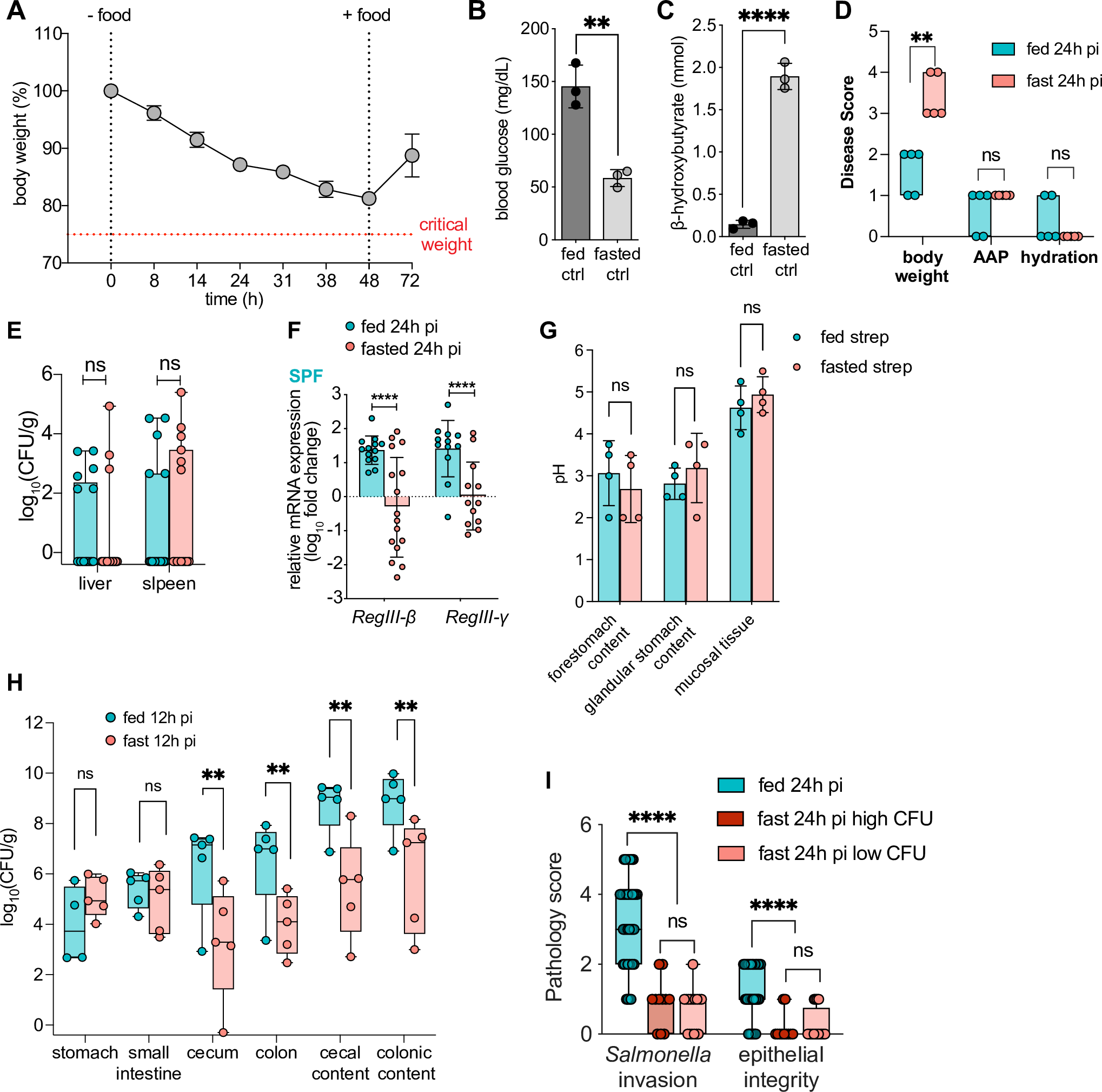

Supplement: S1 Fig — (A) Body weight loss over time during fasting and re-feeding in un-infected control C57BL/6 mice. Critical weight (25% loss of inital body weight) refers to animal ethics protocol limit that cannot be exceeded during any experiment. (n = 7) (B) Glucose levels in mouse whole blood in control un-infected ad libitum fed or 48h fasted mice. (C) β-hydroxybutyrate levels in mouse serum in control un-infected ad libitum fed or 48h fasted mice. (D) Disease/Health Score assessing impact of 24h infection ± fasting on mice. AAP = Activity, Appearance, Posture. Detailed information on scoring matrix can be found in the Materials and Methods section. (E) S. Typhimurium CFU per g liver or spleen tissue 24h p.i. ± 48h of fasting. (see Fig 1A for experimental timeline). (F) qPCR analysis of two antimicrobial-peptide genes in S. Typhimurium-infected mouse ceca expressed as fold change over fed ctrl. Fed ctrl refers to uninfected ad libitum fed C57BL/6 SPF mice. (G) pH of stomach content and tissue of uninfected mice 24h after oral strep treatment ± 24h of fasting. (H) S. Typhimurium CFU per g tissue 12h p.i. ± 36h of fasting. (I) Invasion score quantifying S. Typhimurium presence in IEC by analyzing immunofluorescently stained cecal sections at 24h p.i. Data identical to Fig 1I–but fasting group was split up into mice with high (≥ CFU 106) or low (CFU 103–105) pathogen burdens. **** p < 0.0001, ** p < 0.01, Significance levels calculated by unpaired Student’s t test (B,C), multiple t-test (G), Mann-Whiteney-Wilcoxon test (D, E, H, I)–each with Holm-Sidak correction, two-way ANOVA with Tukey post test (F). Error bars shown as ± SD for (A-C, F-G), and as box plot with min-max whiskers (D, E, H, I). (TIF) [file ppat.1009719.s001.tif]

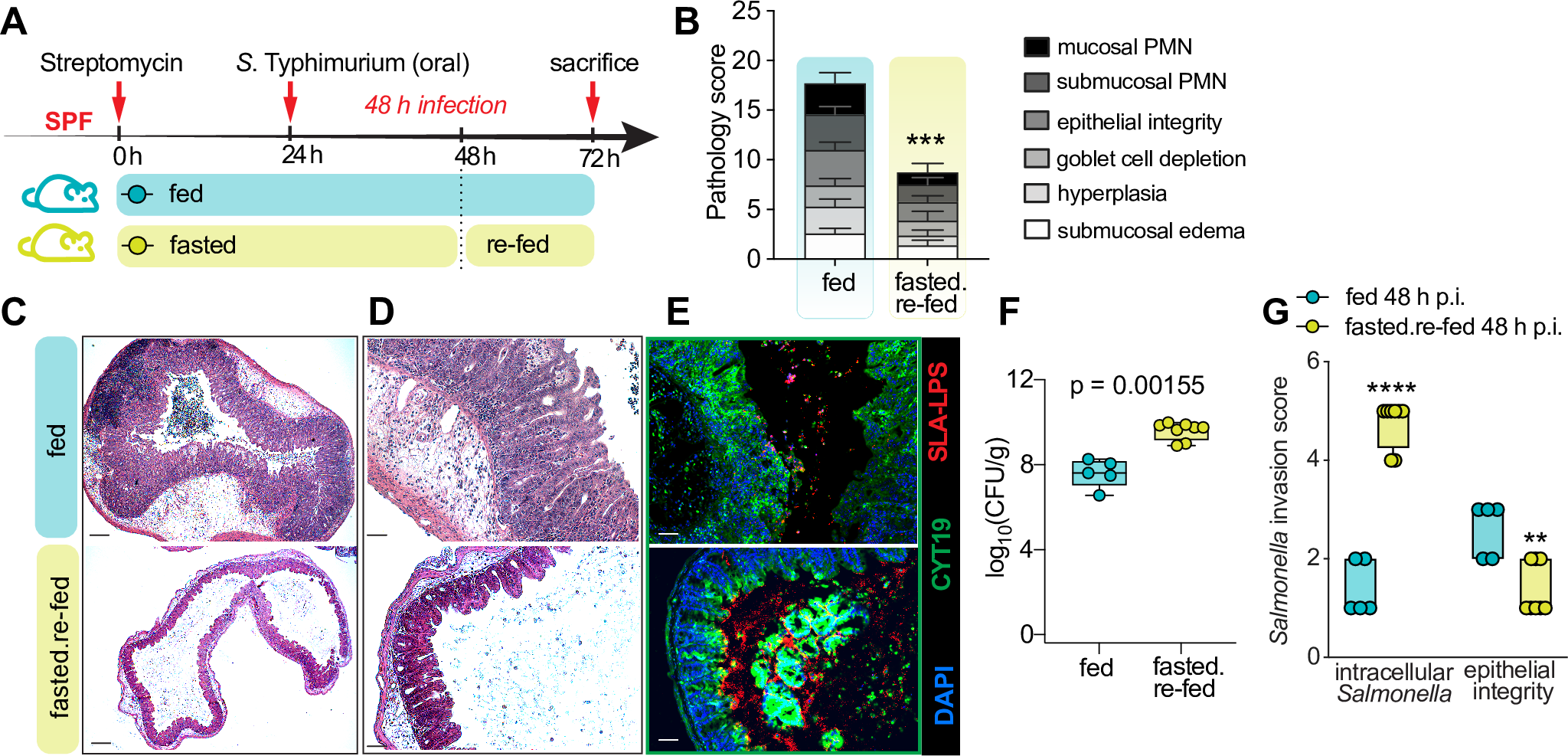

Supplement: S2 Fig — (A) Experimental timeline of infection and diet regimen. Streptomycin-pretreated SPF mice were orally gavaged with ~ 2.5 × 10^6^ colony-forming units (CFU) S. Typhimurium and sacrificed 48h p.i. Mice were either fed throughout the whole experiment (blue), or fasted for the first 48h and re-fed the following 24h of the experiment (green). (C, D) Representative H&E-stained cecal sections of mice at 48h p.i. with S. Typhimurium (see S2A Fig for experimental timeline). Scale bar 200 μm (DC), 50 μm (D). (B) Histopathological analysis of cecal tissue H&E sections (as shown in C-D, see Materials and Methods for scoring criteria). Agreement among raters ensured by Kendall’s coefficient of concordance WT = 0.8385 (n ≥ 7). (E) Representative immunofluorescence staining of S. Typhimurium and IEC on paraffin embedded cecal sections at 48h p.i. ± 48 h of fasting. Sections were stained using DAPI to detect DNA (blue), anti-Salmonella-LPS (SLA, red) to visualize S. Typhimurium and anti-cytokeratin 19 (CYT19, green) to stain IEC. Scale bar 50 μm. (F) Enumeration of S. Typhimurium CFU per g cecal tissue and stool (combined) of mice at 48h p.i. (G) Invasion score quantifying S. Typhimurium presence in IEC by analyzing immunofluorescently stained paraffin-embedded cecal sections at 48h p.i. ± 48h of fasting shown in (E) (see Materials and Methods for scoring criteria). Agreement among raters ensured by Kendall’s coefficient of concordance WT = 0.939 (n ≥ 5). For (B), (F) and (G) data from multiple independent experiments were pooled. **** p < 0.0001, *** p < 0.001, ** p < 0.01, * p < 0.05, ns = not significant. Significance levels calculated by unpaired Student’s t test (B), Mann-Whitney-Wilcoxon test with Holm-Sidak correction (F, G). Error bars shown as ± SD (B); box plot with min-max whiskers (F, G). (TIF) [file ppat.1009719.s002.tif]

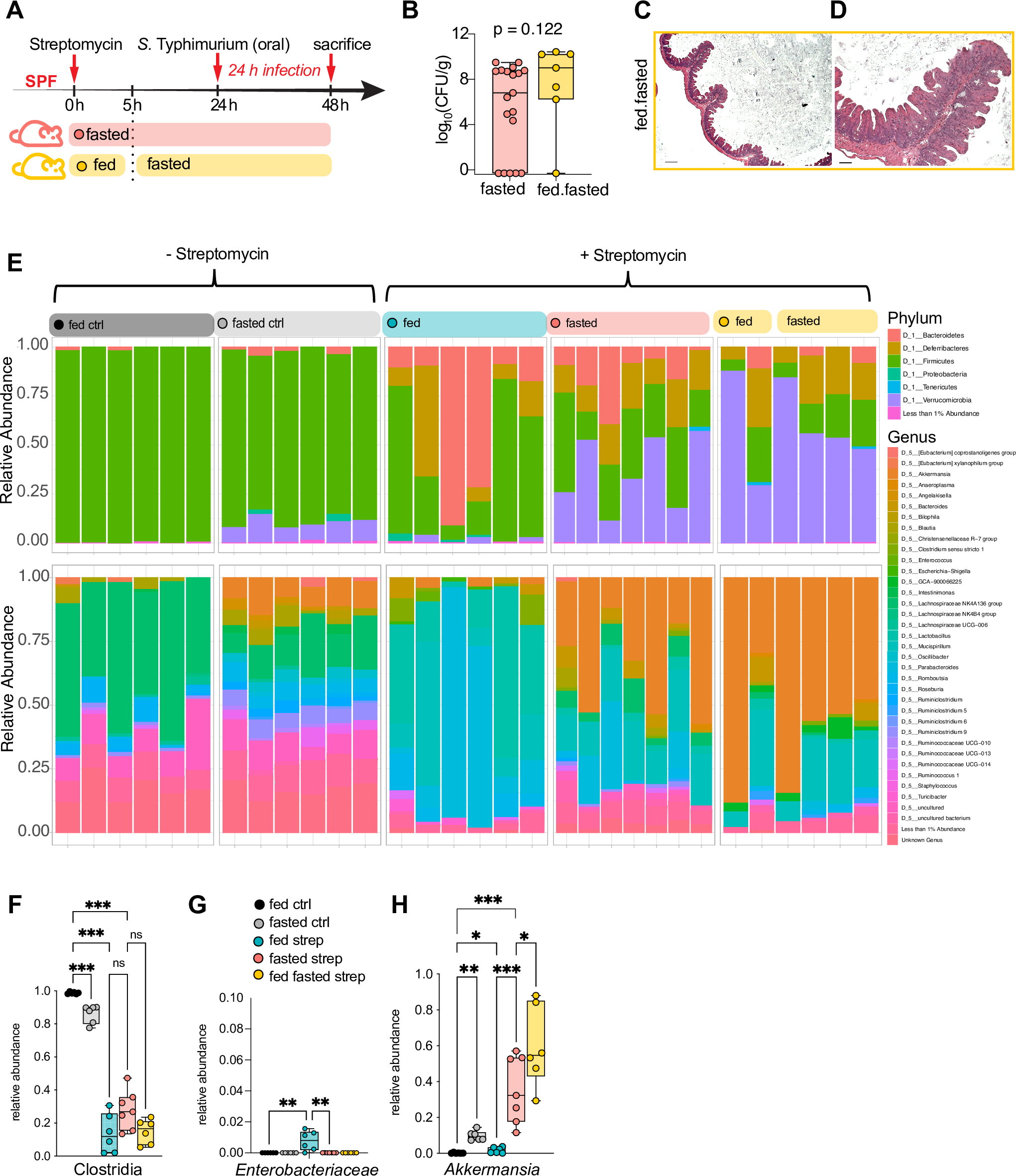

Supplement: S3 Fig — Fasting does not affect the efficacy of streptomycin but changes cecal microbiome composition (A) Experimental timeline of streptomycin treatment and fasting/infection regimen. SPF C57BL/6 mice were orally gavaged with 20 mg of streptomycin and fasted for the next 24h. A second group was given streptomycin, fed for another 5h and fasted for the following 19h. Mice were then orally infected with ~ 2.5 × 10^6^ CFU S. Typhimurium and sacrificed at 24h p.i. (B) Enumeration of S. Typhimurium CFU per g cecal tissue and stool (combined) of mice at 24h p.i. (see S3A Fig for experimental timeline, data for fasted mice is the same as shown in Fig 1J). Data pooled from multiple independent experiments and shown as box plot with min-max whiskers. Significance levels determined by multiple Mann-Whitney-Wilcoxon test with Holm-Sidak correction. (C,D) Representative H&E stained cecal sections of fed.fasted mice (yellow group) at 24h p.i. (see S3A Fig for experimental timeline). Scale bar 200 μm (C), 50 μm (D). (E) Relative abundance of bacteria from the cecal content of SPF mice (for experimental timeline see Fig 3A) on phylum and genus levels. Taxa that were less than 1% abundant were grouped together for better visibility.(F-H) Relative abundance of Clostridia (F), Enterobacteriaceae (G) and Akkermansia (H) across groups. (TIF) [file ppat.1009719.s003.tif]

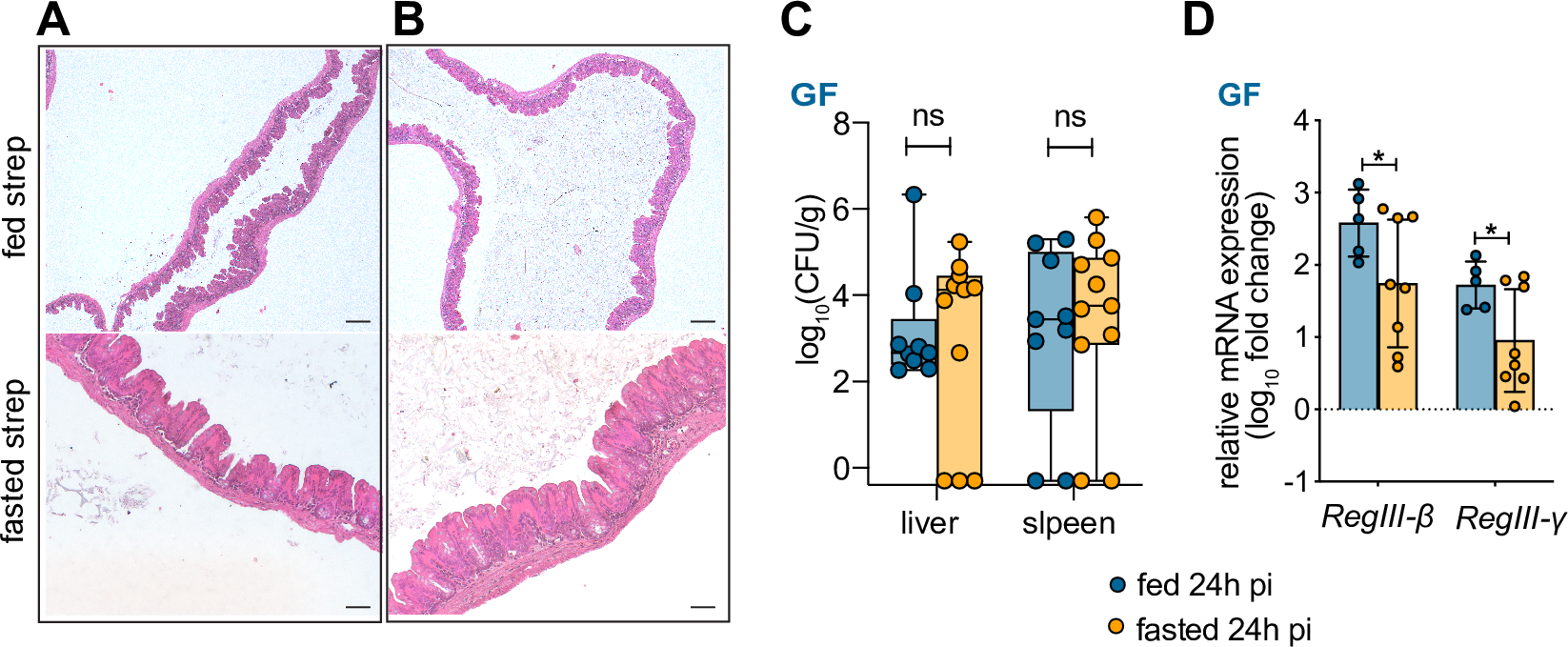

Supplement: S4 Fig — Fasting protects germfree mice from gastroenteritis but not from epithelial cell invasion (A,B) Representative H&E-stained cecal sections of GF mice 24h post-streptomycin treatment, uninfected ± 24h of fasting. Scale bar 200 μm (C), 50 μm (D). (C) S. Typhimurium CFU per g liver or spleen tissue 24h p.i. ± 48h of fasting in GF mice. (see Fig 4A for experimental timeline). (D) qPCR analysis of two antimicrobial-peptides in S. Typhimurium-infected mouse ceca expressed as fold change over fed ctrl. Fed ctrl refers to uninfected ad libitum fed C57BL/6 GF mice. For (C) and (D) data from multiple independent experiments were pooled. * p < 0.05, ns = not significant. Significance levels calculated by multiple Mann-Whitney-Wilcoxon test with Holm-Sidak correction (C), or two-way ANOVA with Tukey post test (D). Error bars shown as ± SD (C), box plot with min-max whiskers (D). (TIF) [file ppat.1009719.s004.tif]

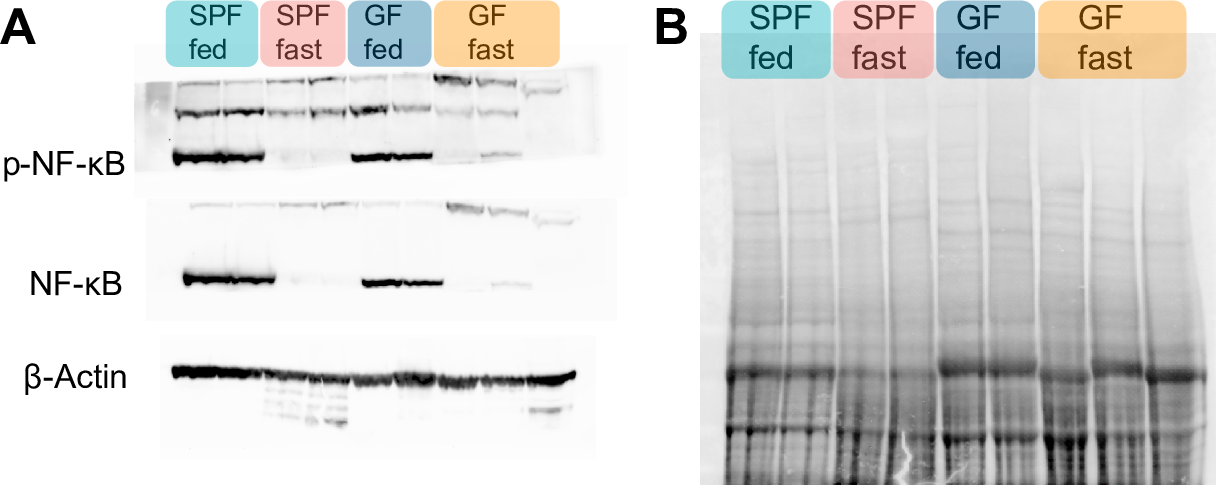

Supplement: S5 Fig — (A) Representative immunoblot detecting phospho and total NF-κB p65/RelA protein in whole cecal tissue lysates of SPF and GF mice at 24h p.i. with S. Typhimurium ± 48h of fasting. Lanes represent data from individual mice. Shown here is the whole PVDF membrane (cut into respective sizes to probe for target), cropped bands of interest are shown in Fig 5F. (B) Total protein stain of whole PVDF membrane (used for Figs 5F and S5A) additionally confirming comparabale protein loading in all lanes. (TIF) [file ppat.1009719.s005.tif]

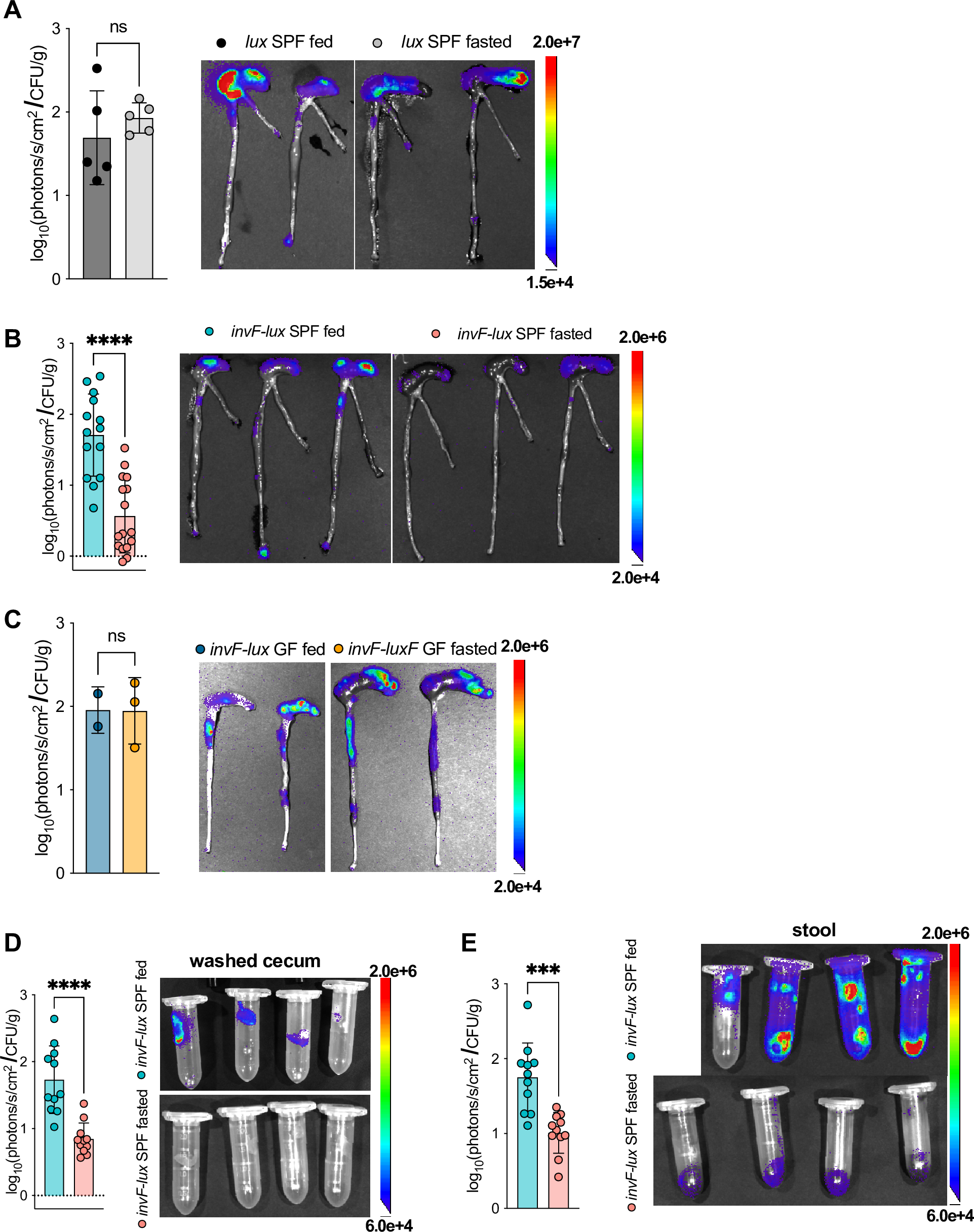

Supplement: S6 Fig — (A-E) Streptomycin-pretreated mice were orally infected with the respective S. Typhimurium strain, at a dose of ~ 2.5 × 10^8^ CFU. Mice were euthanized and intestines imaged at 15h p.i. ± 39h of fasting. Bioluminescence signals were measured in logarithmic units of light (photons/s/cm2/sr), divided by log10CFU of Salmonella pathogen burden. Resulting ratios are expressed as log10 of percent change fed over fasted. (A) Representative macroscopic ex vivo images and corresponding quantification of bioluminescence signal in SPF fed and fasted mouse intestines infected with a Salmonella strain expressing the Photorhabdus luminescens lux operon on the chromosome (ST lux). Images show magnitude of bioluminescence signal corresponding to presence of total Salmonlla numbers. (B,C) Representative macroscopic ex vivo images and corresponding quantification of SPF (B) and GF (C) mouse intestine showing magnitude of bioluminescence signal corresponding to expression of the Salmonella SPI-1 invF-luxCDABE transcriptional fusion. Images in (C) are the same data as shown in Fig 6B. (D,E) Representative images and corresponding quantification of SPF mouse washed cecum (D) and stool (E) bioluminescence signal imaged within Eppendorf tubes. Bioluminescence signal corresponding to expression of the Salmonella SPI-1 invF-luxCDABE transcriptional fusion. For (A, B, D, E) data from multiple independent experiments were pooled. **** p < 0.0001, *** p < 0.001, ns = not significant. Significance levels calculated by unpaired Student’s t test. Error bars shown as ± SD. (TIF) [file ppat.1009719.s006.tif]

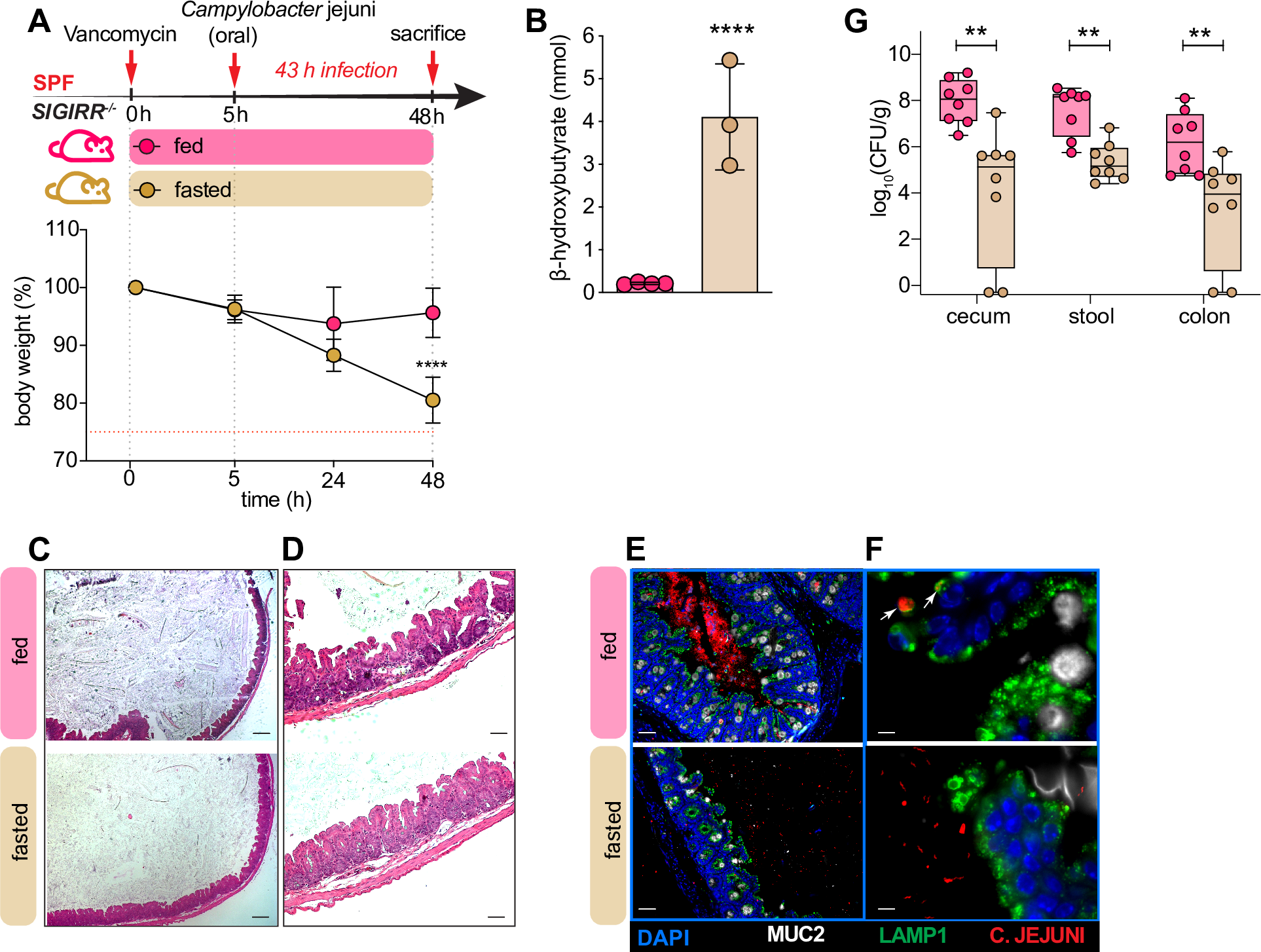

Supplement: S7 Fig — (A) Experimental timeline of infection and fasting regimen and concurrent body weight loss. SPF Sigirr-/- mice were orally gavaged with 5 mg vancomycin and 5h later orally infected with ~ 1 × 10^7^ CFU C. jejuni and sacrificed 43h p.i. Mice were either fed (pink) or fasted (brown) throughout the whole experiment. (B) β-hydroxybutyrate levels in mouse serum in fed or 48 h fasted Sigirr-/- mice 43h p.i. with C. jejuni. (C, D) Representative H&E-stained cecal sections of mice at 43h p.i. with C. jejuni (see S7A Fig for experimental timeline). Scale bar 200 μm (C), 50 μm (D). (E, F) Representative immunofluorescence staining of C. jejuni on paraffin embedded cecal sections at 24h p.i. ± 48h of fasting. Sections were stained using DAPI to detect DNA (blue), anti-Mucin-2 to stain goblet cells filled with mucus, anti-C. jejuni (red) and anti-LAMP-1 (LAMP1, green) to stain for bacteria in Campylobacter-containing vacuoles (indicated by white arrows). Scale bar 50 μm (E), 10 μm (F). (G) Enumeration of C. jejuni CFU per g tissue or stool at 43h p.i. For (A) and (G) data from multiple independent experiments were pooled. **** p < 0.0001, ** p < 0.01. Significance levels calculated by unpaired Student’s t test (B), multiple t-test (A) or Mann-Whitney-Wilcoxon test (G) with Holm-Sidak correction. Error bars shown as ± SD (A,B), box plot with min-max whiskers (G). (TIF) [file ppat.1009719.s007.tif]
